# Computational and Biochemical Analysis of the *Xanthomonas* Effector AvrBs2 and Its Role in the Modulation of *Xanthomonas* Type Three Effector Delivery

**DOI:** 10.1371/journal.ppat.1002408

**Published:** 2011-12-01

**Authors:** Bingyu Zhao, Douglas Dahlbeck, Ksenia V. Krasileva, Richard W. Fong, Brian J. Staskawicz

**Affiliations:** 1 Department of Plant and Microbial Biology, University of California, Berkeley, California, United States of America; 2 Department of Horticulture, Virginia Polytechnic Institute and State University, Blacksburg, Virginia, United States of America; Ohio State University, United States of America

## Abstract

Effectors of the bacterial type III secretion system provide invaluable molecular probes to elucidate the molecular mechanisms of plant immunity and pathogen virulence. In this report, we focus on the AvrBs2 effector protein from the bacterial pathogen *Xanthomonas euvesicatoria* (*Xe*), the causal agent of bacterial spot disease of tomato and pepper. Employing homology-based structural analysis, we generate a three-dimensional structural model for the AvrBs2 protein and identify catalytic sites in its putative glycerolphosphodiesterase domain (GDE). We demonstrate that the identified catalytic region of AvrBs2 was able to functionally replace the GDE catalytic site of the bacterial glycerophosphodiesterase *BhGlpQ* cloned from *Borrelia hermsii* and is required for AvrBs2 virulence. Mutations in the GDE catalytic domain did not disrupt the recognition of AvrBs2 by the cognate plant resistance gene *Bs2*. In addition, AvrBs2 activation of Bs2 suppressed subsequent delivery of other *Xanthomonas* type III effectors into the host plant cells. Investigation of the mechanism underlying this modulation of the type III secretion system may offer new strategies to generate broad-spectrum resistance to bacterial pathogens.

## Author Summary

The bacterial pathogen *Xanthomonas euvesicatoria* (*Xe*) is the causal agent of bacterial leaf spot disease of pepper and tomato. This pathogen is capable of delivering more than 28 effector proteins to plant cells via the type three secretion and translocation system (TTSS). The AvrBs2 protein is a TTSS effector of *Xe* with a significant virulence contribution that depends on a conserved glycerolphosphodiesterase (GDE) domain. Additionally, activation of the resistance protein Bs2 by AvrBs2 modulates the TTSS of *Xe* and suppresses the subsequent delivery of TTSS effectors.

## Introduction

Plants have evolved sophisticated innate immune systems to counter the attack of various microbial pathogens through a combination of diverse molecular mechanisms [1]. Plant innate immunity is controlled by two overlapping signaling pathways. The first pathway, PAMP-Triggered Immunity (PTI), is a basal defense response that is triggered by the recognition of pathogen-associated molecular patterns (PAMPs) through a set of specialized plant extracellular receptor kinase proteins [2]–[5]. Plants use PTI to suppress the growth of non-pathogens. However, successful bacterial pathogens can interfere with PTI via effector proteins that are delivered into plant cells through the type three secretion and translocation system (TTSS). Many bacterial TTSS effectors have identified virulence functions that modulate the pathways involved in PTI, making the plants more susceptible to the proliferation of microbial pathogens [1]. Most of these TTSS effector proteins are not homologous, and the majority have no obvious biochemical function, although a few have been shown to have enzymatic activity [6]–[9]. Characterizing the biochemical functions of pathogen effectors and identifying the plant targets of each effector will shed light on bacterial pathogenesis and plant immunity. In response to effector proteins, plants have evolved a second layer of defense signaling pathways controlled by resistance genes (*R* genes). The plant R proteins directly or indirectly recognize the bacterial TTSS effectors and initiate effector-triggered immunity (ETI) [10]. This response is often a localized, programmed cell death-related defense response, also known as the hypersensitive reaction (HR) [11]. Despite intensive study of the molecular mechanisms of PTI and ETI, the interplay between these two primary defense mechanisms remains elusive [12], [13].

The TTSS machinery of phytopathogenic bacteria encoded by the clustered *hrp* (*h*ypersensitive *r*eaction and *p*athogenicity) genes is essential for the delivery of effectors to the interior of the plant cell [14]. Mutations in the pathogen that block the TTSS will subsequently prevent the translocation of the type III effectors and impair the virulence of the pathogen on host plants [14]–[16]. Therefore, the TTSS plays a critical role in bacterial pathogenesis. The translocation of TTSS effectors can be quantitatively measured by monitoring adenylate cyclase enzyme activity in plant cells by fusing the effector protein with the calmodulin-dependent adenylate cyclase domain (*Cya*) of *Bordetella pertussis* cyclolysin [17], [18]. Despite intensive characterization of the TTSS in model bacterial pathogens, including several *Pseudomonas* and *Xanthomonas* species, detailed information describing the establishment and regulation of the TTSS is still missing. It is also not clear if plants have evolved defense mechanisms that can recognize the establishment of bacterial TTSS. However, a recent report demonstrated that PTI of the host plant can inhibit the injection of bacterial type III effectors [19], suggesting that the suppression of TTSS may contribute to the plant immunity.

*Xanthomonas euvesicatoria* (*Xe*) is the causal agent of bacterial leaf spot disease of pepper and tomato, which can deliver more than 28 TTSS effectors into plant cells [20], [21]. One type III effector AvrBs2 is highly conserved not only in *Xe* strains but also in many other *Xanthomonas* pathovars that cause disease in a wide range of crops [22], [23]. The presence of *avrBs2* in many of these pathogens makes a significant contribution toward their virulence [22]. Previous analyses have determined that the *avrBs2* gene encodes a protein containing a domain homologous to the *E. coli* glycerolphosphodiesterase (GDE) and the agrocinopine synthase (ACS) of *Agrobacterium tumefaciens*. However, it has not been shown whether AvrBs2 possesses GDE or ACS enzyme activity and whether such activity is relevant to AvrBs2 function [23], [24].

Pepper plants (*Capsicum annuum*) carrying the bacterial leaf spot disease resistance gene (*Bs2*) are resistant to strains of *Xe* that contain AvrBs2. This host-pathogen interaction results in a resistance response that inhibits the growth of *Xe* [22]–[25]. The *Bs2* gene has been isolated by map-based cloning and encodes a protein that belongs to the largest class of plant disease resistance proteins. The protein contains a central putative nucleotide-binding site (NBS) and a carboxyl-terminal leucine-rich repeat (LRR) region [25]. Bs2 has been shown to associate with the molecular chaperone SGT1 through its LRR domain to specifically recognize AvrBs2 and trigger the HR in plants [26]. However, it is still not clear whether Bs2 recognizes AvrBs2 directly or indirectly *in planta*.

In addition to the *Bs2* gene, two other pepper resistance genes, *Bs1* and *Bs3*, have been identified that confer resistance to *Xe* strains carrying the *avrBs1* and *avrBs3* effector genes, respectively [27]. Near-isogenic lines carrying the *Bs1, Bs2,* and *Bs3* genes have been generated by introgression of individual or combinations of *Bs* genes into the susceptible pepper cultivar Early Cal Wonder (ECW) [28], [29]. The *avrBs1* and *avrBs3* genes have also been identified and cloned [23], [30]–[32]. The *Bs1* gene has not been cloned [32], but *Bs3,* which encodes a flavin monooxygenase enzyme, has recently been isolated from the pepper genome [33].

In this study, the pepper and *Xe* pathosystem is used to study the interaction between Bs2 and AvrBs2. We demonstrate that the catalytic sites of the putative GDE domain of AvrBs2 are under purifying selection, and that the GDE catalytic sites are required for AvrBs2 virulence function but not the activation of Bs2. Although we were unable to demonstrate the GDE enzymatic activity using purified, full-length AvBs2, we determine that the AvrBs2 GDE catalytic site could functionally replace the GDE catalytic site of *Bh*GlpQ (*Borrelia hermsii)* [34]. We also identify a minimum domain of AvrBs2 that included the GDE homologous region and a carboxyl Bs2 activation domain. Therefore, we are able to genetically separate the virulence function of AvrBs2, which is dependent on its GDE catalytic site, from the Bs2 activation, which is independent of the GDE catalytic site.

Finally, we describe a novel plant disease resistance phenotype related to the AvrBs2/Bs2 host-pathogen interaction. When AvrBs2 activates the Bs2 R gene function, the TTSS is reduced in the delivery of effectors to the plant host. Investigation of the mechanism of the AvrBs2 virulence function and TTSS suppression during its recognition by Bs2 could offer new strategies to generate broad-spectrum resistance to the *Xe* bacterial pathogen.

## Results

### Computational and biochemical evidence that AvrBs2 contains an active GDE catalytic domain

**Figure 1:**
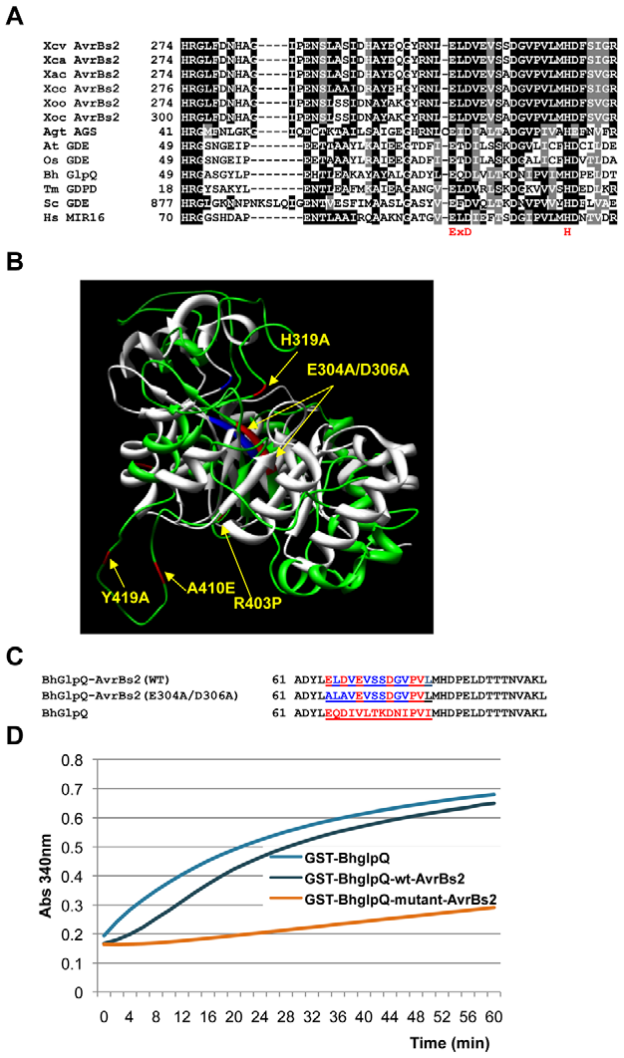
Structural model of the AvrBs2 phosphodiesterase (GDE) domain and in vitro GDE activity. **A**. CLUSTALW alignment of the GDE domain shared by Xe AvrBs2 (amino acids 274 to 328) and its homologs from Xanthomonas pathogens of tomato, euvesicatoria (Xe); alfalfa, campestris pv. alfalfa (Xca); citrus, axonopodis pv. citri (Xac); cabbage, campestris pv. campestris (Xcc); and rice, oryzae pv. oryzae (Xoo) and oryzae pv. oryzicola (Xoc). Also includes selected GDE (orputative GDE) proteins from planta (AtGDE and OsGDE), animala (HsMIR16), fungi (ScGDE1), and other bacteria (Tm GDPD, BhGlpQ and AgtACS). The conserved putative catalytic sites of the human GDE, HsMIR16 are in red. **B**. The three-dimensional structural model for AvrBs2 (amino acids 274 to328) using the solved crystal structure of TmGPDO (1o1z A) as a template. TmGPDO 1o1z A is displayed in gray, and AvrBs2 is in green. The GDE catalytic sites ExD and H are blue in 1o1z A and red in AvrBs2. Shown in yellow are the amino acid mutations in the AvrBs2 putative catalytic sites and three additional sites that disrupt AvrBs2 activation of Bs2. **C**. Multiple sequence alignment of a 23 amino acid area of the GDE, BhGlpQ, catalytic site (amino acid 61–84) for two chimeric BhGlpQ proteins with the underlined sequences of the GDE catalytic site from either wild-type AvrBs2or AvrBs2 GDE mutants. **D**. In vitro GDE activity. The GDE enzyme activity of purified GST:BhGlpQ (positive control), GST:BhGlpQ-AvrBs2-WT, and BhGlpQ-AvrBs2-E304A/D306A were analyzed using an indirect coupled enzyme assay. A higher absorbance indicates increased GDE enzyme activity.

Previous characterization of AvrBs2 (YP 361783) from *Xe* revealed a domain [amino acids (aa) 280 to 340] with homology to a bacterial GDE [23]. To further characterize this *Xe* AvrBs2 domain, we searched the current GenBank database with the BLASTP program using the full-length AvrBs2 protein as a query. This search allowed us to compile remote homologs from plants, animals, fungi, and bacteria that contain GDE domains homologous to AvrBs2. In Figure 1A, selected GDE (or putative GDE) proteins from plants [*At*GDE (NP_177561)] and *Os*GDE [(AP003274)], human [*Hs*MIR16 (NP_057725)], fungi [*Sc*GDE1 (NP_015215)], and bacteria [*Tm*GDPD (TM1621) of *Thermotoga maritima, Bh*GlpQ (ADD63790) from *Borrela hermsii*, and *Agt*ACS (AAO15364) from *Agrobacterium tumefaciens*] aligned with the GDE domain of AvrBs2 (aa 274 to 328) are shown. Several AvrBs2 homologs from *Xanthomonas* pathogens of tomato, *euvesicatoria* (*Xe*) (YP_361783); alfalfa, *campestris* pv. *alfalfae* (*Xca*) citrus, *axonopodis* pv. *citri* (*Xac*) (NP_640432); cabbage, *campestris* pv. *campestris* (*Xcc*) (NP_635447); and rice, *oryzae* pv. *oryzae* (*Xoo*) (YP_449177) or *oryzae* pv. *oryzicola* (*Xoc*) (ZP_02241238) were included in the alignment. The overall sequence identity between AvrBs2 and the different GDEs in this region was approximately 33% (with >37% sequence similarity) (Figure 1A) [35]. The putative GDE domain in AvrBs2 aligned well with the glycerophosphodiester phosphodiesterase (GdPd) protein from *Thermotoga maritima,* for which the three-dimensional crystal structure had been previously determined (PDB ID: 1O1Z) [36]. The GDE domains of AvrBs2 and *Tm*Gdpd share 60% amino acid sequence similarity and 47% identity. The high amino acid sequence similarity between the GDE domains of AvrBs2 and *Tm*Gdpd predicts that these two proteins will have similar three-dimensional structures.

A homology-based modeling method was employed to generate a three-dimensional structural model for AvrBs2 (aa 274 to 328) using the solved crystal structure of TmGdpd as a template [36], [37]. The resulting three-dimensional structural model of AvrBs2 closely matched the solved crystal structure of Tm 1o1z A (Figure 1B). Both structures consist of two antiparallel beta-sheets capped by nine putative alpha-helices. Recently, GDE enzyme activity and the putative catalytic sites of the human GDE (*Hs*MIR16) have been characterized [38], [39]. Point mutations in the GDE catalytic sites (E97A, D99A, and H112A) in *Hs*MIR16 eliminated GDE enzyme activity [38], [39]. The putative catalytic sites of *Hs*MIR16 are conserved in all of the GDE homologs, including the six AvrBs2 homologs (Figure 1A). In the three-dimensional structural model of AvrBs2, the catalytic sites are present in regions of high structural homology between the two proteins (TmGdpd in blue and AvrBs2 in red), which suggests that AvrBs2 utilizes the same residues for enzymatic function (Figure 1B).

To investigate whether the AvrBs2 protein possesses GDE enzyme activity, both the wild type and the catalytic mutants of *avrBs2* were expressed in *E. coli* as GST-AvrBs2 fusion proteins. The fusion proteins were assayed for GDE enzyme activity using a method that was originally adapted for *E. coli* and *Borrelia* GDEs, with glycerophosphocholine as a substrate [40], [41]. However, we were unable to detect GDE enzyme activity of AvrBs2 with this substrate. Because the GDE catalytic sites of the *Bh*GlpQ enzyme were conserved with predicted catalytic sites in AvrBs2 (Figure 1A), we hypothesized that if we replaced the core GDE catalytic site of the active *Bh*GlpQ enzyme [41] (24 amino acids) with the putative GDE catalytic site of AvrBs2, we might be able to detect enzyme activity with glycerophosphocholine substrate *in vitro*. To test this possibility, the GDE catalytic site of *Bh*GlpQ was replaced with either the wild-type AvrBs2 catalytic site or a GDE catalytic site mutant (E304A/D306A) (Figure 1C). The GDE enzyme activities of purified GST:*Bh*GlpQ (positive control), GST:*Bh*GlpQ-AvrBs2-WT, and GST:*Bh*GlpQ-AvrBs2-E304A/D306A were analyzed using an indirect coupled enzyme assay [41]. The higher light absorbances at 340 nm for GST:*Bh*GlpQ (positive control) and GST:*Bh*GlpQ-AvrBs2-WT compared to the inactive GST:*Bh*GlpQ-AvrBs2-E304A/D306A indicated that AvrBs2 had a functional GDE catalytic site (Figure 1C and 1D).

### The GDE catalytic sites of AvrBs2 are required for virulence but not for activation of the Bs2-specified disease resistance signaling pathway

**Figure 2:**
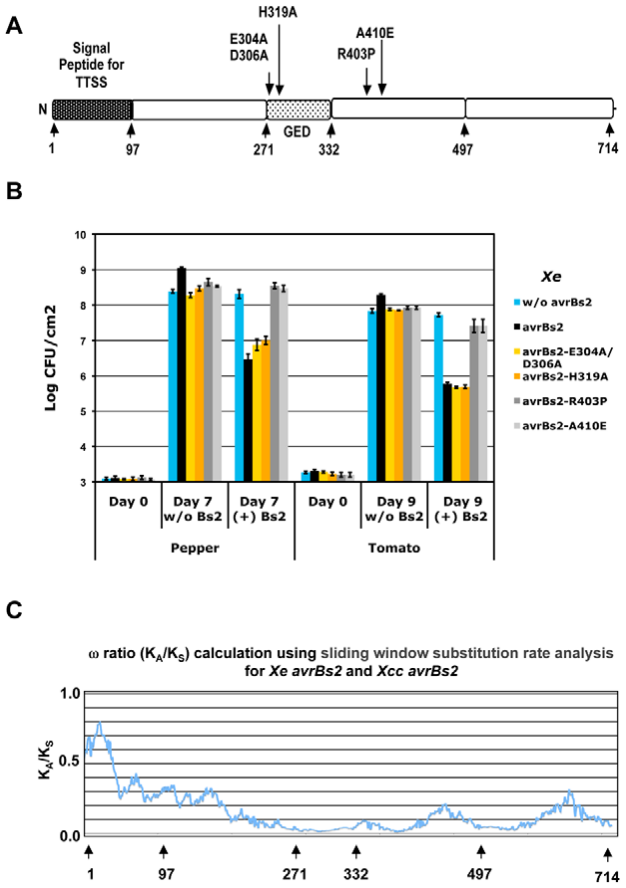
The GDE catalytic sites are required for AvrBs2 virulence function. **A**. The map of the AvrBs2 coding region with numbers representing amino acid positions. AvrBs2 mutations for the GDE catalytic site and the AvrBs2 mutations from *Xe* field strains isolated from diseased BS2 pepper. **B**. In planta pathogen growth assay for *Xanthomonas* strains GM98-38 *X*e (w/o *avrBs2*), GM 98-38-1 *Xe* (*avrBs2*), and GM98-38-1 exchange mutants for the putative GDE catalytic site *Xe* (*avrBs2*-E304A/D306A) and Xe (avrBS2-H319A) and two previously published control exchange mutants *Xe* (*avrBs2*-R403P) and Xe (avrBs2-A410E). Host plants include the near-isogenic pepper (w/o *Bs2*) and pepper (*Bs2*) along with the tomato line VF36 (w/o *Bs2*) and the previously published transgenic line VF36 (*Bs2*). Student t-test was used to compare different growth assays with the most virulent case (Xe (*avrBs2*) on non*-Bs2* plants) for both Pepper and Tomato hosts; p-values were <0.01 for all other combination when compared to (Xe (*avrBs2*) on non*-Bs2* plants). The *Xe* strains carrying AvrBs2 mutations for the GDE catalytic site still activate full *Bs2* resistance but do not maintain the full virulence in the absence of *Bs2*. There is a corresponding wild type HR brown necrosis phenotype for *Bs2* pepper inoculated with high-density suspensions (2×10^8^ CFU/ml) for these two *Xe* mutant strains (Supplemental Figure S1). The *Xe* strains carrying AvrBs2 mutations from *Xe* field strains isolated from diseased *Bs2* pepper grow to similar levels in the presence or absence of Bs2. There is a corresponding loss of the HR brown necrosis phenotype for *Bs2* pepper inoculated with high-density suspensions of Xe avrBs2-R403P and only a weak HR brown necrosis phenotype for Xe avrBs2-A410E (Supplemental Figure S1). **C**. Plot of amino acid substitution rate analysis using a sliding window calculation of non-synonymous (K_A_) and synonymous (K_s_) changes between *avrBs2* homologs from *Xe* and *Xcc*. The K_A_/K_s_ ratios less than 0.5 indicate that much of AvrBs2 is under purifying selection, including the region homologous to GDE that is required for AvrBs2 virulence function. The numbers below the plot represent amino acid positions.

To test whether the GDE catalytic site of AvrBs2 is important for *Xe* virulence in susceptible bs2 plants or for Bs2 disease resistance activation, we mutated the GDE catalytic sites E304A, D306A and H319A by site-directed mutagenesis of the wild-type *avrBs2* gene (Figure 2A). We replaced the chromosomal copy of *avrBs2* in strain *Xe* GM98-38-1 with various *avrBs2* mutants by homologous recombination. The effects of these mutations on AvrBs2 virulence function and/or Bs2-activation were evaluated by in planta bacterial growth assays in near-isogenic pepper and tomato lines with and without the R gene *Bs2* (Figure 2B). In pepper and tomato lines without *Bs2*, the *Xe* strain with wild-type *avrBs2* was more virulent and grew approximately five-fold higher than the null strain *Xe* without *avrBs2* (Figure 2B). The *Xe* strains with mutations in GDE domain (E304A/D306A and H319A) lost AvrBs2 virulence function and were similar to the null strain *Xe* without *avrBs2* (Figure 2B). However, on near-isogenic pepper and transgenic tomato lines with *Bs2* [25], *Xe* strains carrying the AvrBs2 GDE mutants were still able to activate Bs2-based resistance, similar to the *Xe* strain carrying wild-type *avrBs2* (Figure 2B). These results demonstrate that the putative GDE catalytic sites of *avrBs2* are required for its virulence function but not for recognition by Bs2.

Additionally, we tested two control *Xe* strains that contain point mutations (R403P and A410E) [24] that evade Bs2 activation while maintaining most of the virulence functions of AvrBs2 (Figure 2A). Similar to previously reported results in pepper plants without *Bs2* [24], these mutants were intermediate in virulence between *Xe* carrying wild-type *avrBs2* and *Xe* without *avrBs2*. However, the mutants were unable to activate *Bs2* resistance in pepper plants containing *Bs2* (Figure 2B).

Another method for assaying the induction of plant immunity is to challenge a plant with a high-density bacterial dose that triggers a macroscopic hypersensitive cell death reaction, or HR response. High-density inoculations (2×10^8^ CFU/ml) of pepper with *Bs2* caused a similar, strong brown necrosis with the *Xe* strain with wild-type *avrBs2* and the *Xe* strains with *avrBs2* GDE mutations (E304A/D306A and H319A) (Supplemental Figure S1). However, high-density inoculations of pepper plants containing *Bs2* with the *Xe avrBs2* mutant strain (A410E) caused a light brown necrosis, suggesting that this mutant maintained a low level of Bs2 activation capability (Supplemental Figure S1), similar to previously reported [24].

To test whether the GDE mutations had a negative effect on AvrBs2 delivery by *Xe* TTSS, the TTSS effector delivery reporter Cya [18] was utilized to quantitatively measure the translocation of two different AvrBs2 GDE mutant *Xe* effectors. The AvrBs2 GDE mutations caused no reduction of detectable effector delivery (Supplemental Figure S2A). Additionally, the *Xe* (*avrBs2-*Cya) wild type and catalytic site mutant strains were not altered from the non-Cya strains in the activation Bs2 HR (Supplemental Figure S2B).

### The AvrBs2 GDE virulence domain is under strong purifying selection

Demonstrating that the GDE domain of AvrBs2 is required for virulence prompted us to evaluate the natural variations in various *avrBs2* alleles with respect to the evolutionary selection. In addition to the previously published *avrBs2* homologs [(*Xe* in pepper (YP_361783), *Xca* in alfalfa and *Xcc* in cabbage (NP_635447)] [23], three additional uncharacterized homologs of *avrBs2* (*Xanthomonas axonopodis* pv. *citri* [*Xac*] (NP_640432), *Xanthomonas oryzae* pv. *oryzae* [*Xoo*] (YP_449177), and *Xanthomonas oryzae* pv. *oryzicola* [*Xoc*] (ZP_02241238) from newly released genome sequences were aligned using the CLUSTALW program [35]. The overall sequence identity of the different *avrBs2* homologs in *Xanthomonas* was high (>70%). Phylogenetic analysis by maximum likelihood (PAML) software was used to determine which evolutionary model acts on these six homologs of *avrBs2* from different *Xanthomonas* pathovars that have adapted to cause disease in different host plant species [42]. This statistical analysis of nucleotide changes with respect to amino acid changes calculated an average rate of non-synonymous (K_A_) and synonymous (Ks) substitutions per site for all six *avrBs2* homologs. The ratio (ω)  = K_A_/K_s_ measures the difference between the two rates. For neutral amino acid changes or neutral selection, the ω ratio is 1.0. For advantageous amino acid changes or adaptive selection, the ω ratio is >1.0, and for deleterious amino acid changes or purifying selection, the ω ratio is <1.0 [42], [43]. The average ω ratio over all six homologs was estimated to be 0.1534, indicating a strong purifying selection on the *Xanthomonas* pathovars to maintain *avrBs2* for its contribution to pathogenic virulence in a range of different host plant species. In addition, PAML analysis revealed a significant variation in the ω ratio over the length of the *avrBs2* sequence. Sliding window analysis using the SWAKK program [43] was used to determine the distribution of variation in the ω ratio across *avrBs2* from *Xe* and *Xcc*. The low ω over the GDE-virulence region is consistent with purifying selection to maintain the virulence function of *avrBs2* (Figure 2C). Although the ω for the TTSS signal peptide remained below one, there was an increase in ω in this region, possibly associated with differences in TTSS effector delivery for specific *Xanthomonas* pathovars as they infect different host plants (Figure 2C).

### The minimum AvrBs2 domain required for Bs2-activation includes the entire GDE homologous region and an additional C-terminal region

**Figure 3:**
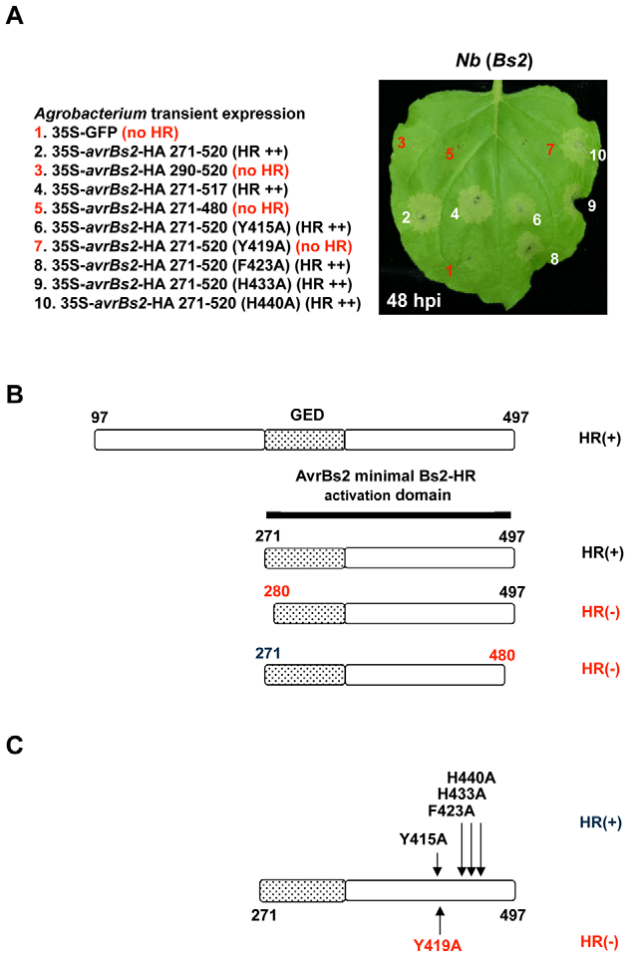
Characterization of the minimal AvrBs2 domain required for Bs2 activation. **A**. Various AvrBs2 deletions and mutations of the minimal AvrBs2 domain required for Bs2 activation are expressed by the 35S promoter transiently on Bs2 *Nicotiana benthamiana* by *Agrobacterium* (48 hpi at 3×108 CFU/ml). **B**. AvrBs2 coding region map with numbers representing amino acid positions. Deletion analysis defined AvrBs2 amino acids 271–520 as the minimal domain for Bs2-activation. In Supplemental Figure S3B we confirm the HR response of these AvrBs2 clones on *Bs2* pepper. Also we detect similar protein expression for all clones using C-terminal HA epitope tags and immunoblot analysis (Supplemental Figure S3A). **C**. Mutational analysis of the C-terminal region of the minimal Bs2-HR activation domain. Single amino acid mutations identify amino acid Y419 as needed for BS2 activation. In Supplemental Figure S3B we confirm the HR response of these AvrBs2 clones on *Bs2* pepper. Also we detect similar protein expression for all clones using C-terminal HA epitope tags and immunoblot analysis (Supplemental Figure S3A).

Having established that the GDE catalytic sites are required for AvrBs2 virulence function but not Bs2-activation, we generated additional deletions of the N-terminus of AvrBs2 to define a minimal region required for Bs2 activation. The deletions were cloned into a binary vector and screened for HR in stable transgenic *Bs2 Nicotiana benthamiana* using *Agrobacterium-*mediated transient expression (Figure 3A). The previously reported [44] *avrBs2* deletion construct (aa 97 to 520) was still able to trigger a *Bs2* HR; the N-terminal deletion (aa 271 to 520) produced a similar result (Figure 3A and 3B). Further deletions at either the amino or the carboxyl terminus of the minimal domain failed to elicit a Bs2-dependent HR. Thus, the fragment (aa 271 to 520) was the minimal region required for Bs2 activation. Interestingly, the minimal Bs2 recognition region included the GDE domain, although an active catalytic site was not required for Bs2 activation. We confirmed the *Agrobacterium-*mediated transient expression HR response of these AvrBs2 mutants on *Bs2* pepper (Supplemental Figure S3B). Also, we detected similar protein expression for all clones using C-terminal HA epitope tags and immunoblot analysis (Figure S3A).

The previously identified AvrBs2 loss-of-Bs2-recognition mutations (R403P and A410E) [24] are within the minimal Bs2 activation domain but are C-terminal to the GDE homologous region. To identify other residues in AvrBs2 near the point mutations of R403P and A410E that might play a role in Bs2 activation, a collection of randomly selected single amino acid mutations in the C-terminal region of the minimal Bs2 activation domain was generated. These fragments were cloned into the same binary vector used for the deletion constructs and used in *Agrobacterium* transient expression experiments. We identified one additional point mutant (Y419A) that had lost the ability to trigger HR (Figure 3A and 3C). In the AvrBs2 three-dimensional structural model (Figure 1B), the Y419A mutation and the two other mutations (R403P and A410E) that also disrupt AvrBs2 activation of Bs2 are located on the loops that do not closely align with the solved crystal structure template (1O1Z). In Supplemental Figure S3A and S3B we confirm the *Agrobacterium-*mediated transient expression HR response of these AvrBs2 mutants on *Bs2* pepper and confirm protein expression.

To further evaluate the role of Y419A, we replaced the wild type *avrBs2* allele of *Xe* with the Y419A mutant by double homologous recombination. The effects of Y419A on AvrBs2 virulence and/or Bs2-activation were evaluated by *in planta* bacterial growth assays (Supplemental Figure S4A). On *Bs2* pepper the *Xe* Y419A mutant strain was intermediate between *Xe* carrying wild-type *avrBs2* and *Xe* without *avrBs2*. High-density inoculations of pepper plants containing *Bs2* with the *Xe avrBs2* mutant Y419A caused a light brown necrosis, suggesting that this mutant maintained a low level of Bs2 activation (Supplemental Figure S4B) similar to the *Xe* mutant A410E (Supplemental Figure S1).

This deletion analysis defined a minimal Bs2 activation domain that included the GDE region, but did not require an active GDE catalytic site. The results of the mutagenesis assays suggest that the critical amino acids for Bs2 recognition are located near the C-terminal end of the minimal Bs2-activation domain. Therefore, the general AvrBs2 structure but not the putative GDE enzymatic activity, was required for Bs2 activation.

### Recognition of the AvrBs2 effector by the Bs2 immune receptor modulates TTSS effector delivery to host plant cells

**Figure 4:**
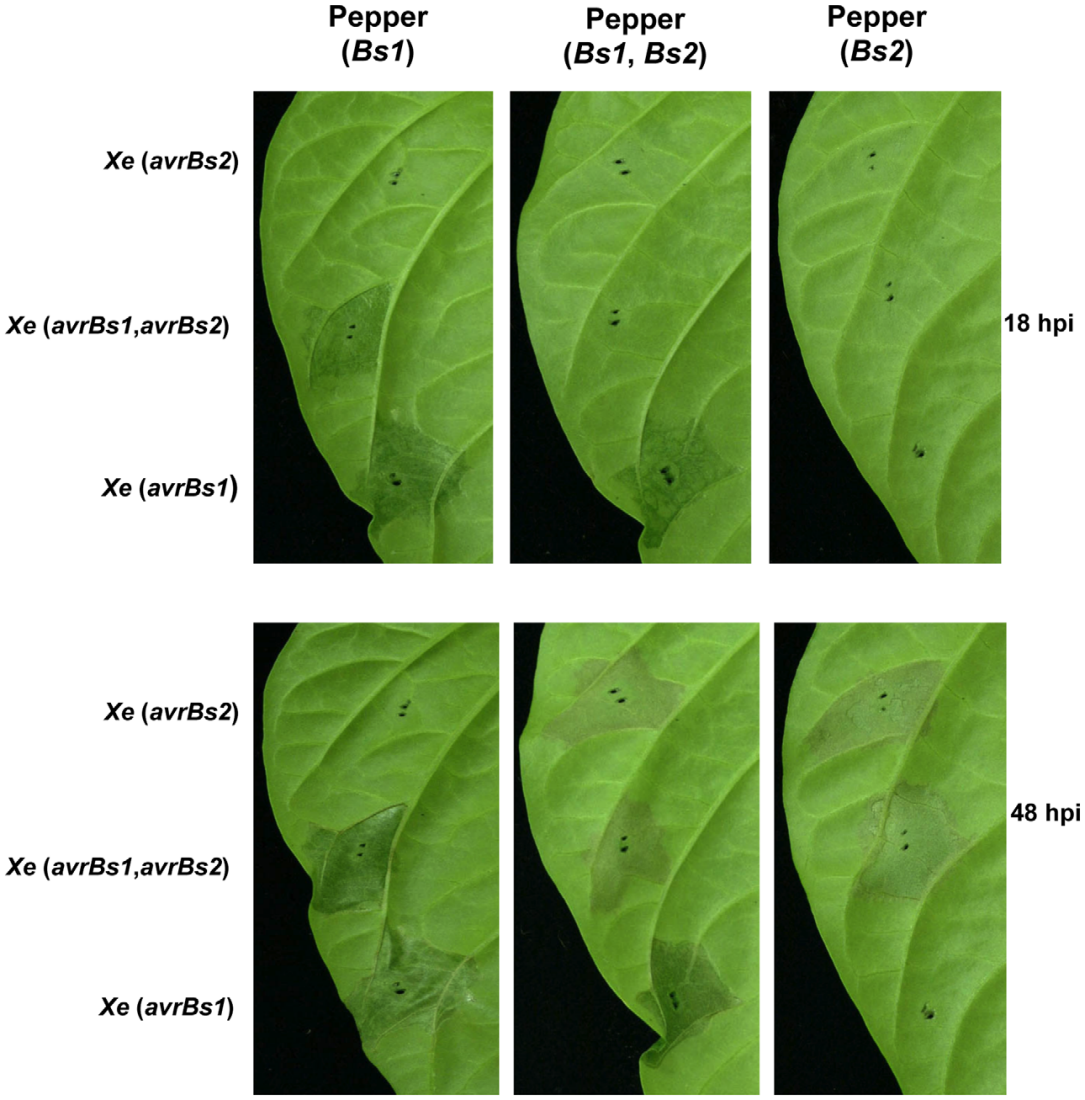
The slower Bs2-HR (48 hpi) from high-density (1.5×10^8^ CFU/ml) inoculation of *Xe* (*avrBs1, avrBs2*) strain was epistatic to the faster Bs1-HR (18 hpi) for pepper (*Bs1, Bs2*). Near-isogenic pepper lines with bacterial spot resistance genes (*Bs1, Bs2* and the combination of *Bs1* and *Bs2*), at 18 and 48 hours post-inoculation (hpi) with the strains *Xe* (a*vrBs1*), *Xe* (*avrBs2*) and Xe (*avrBs1, avrBs2*). When the strain *Xe* (*avrBs1, avrBs2*)) was inoculated on pepper (*Bs1*, *Bs2*) the fast Bs1/AvrBs1 HR was not detected at 18 hpi.

It has long been known that cognate effector/R protein interactions result in a hypersensitive reaction that is specified by the interacting gene pairs. The intensity and the color of the collapsing host tissue and the timing of cell death are specific to the interacting gene pairs. The activation of HR by AvrBs2/Bs2 interactions is slow; macroscopic cell death symptoms appear at 48 hours post-infection (hpi). The *Xanthomonas* effector AvrBs1 activates a rapid Bs1-dependent HR visible at 18 hpi [30]. When we inoculated the *Xe* (*avrBs2*, *avrBs1*) strain delivering both AvrBs1 and AvrBs2 into a pepper line containing both *Bs1* and *Bs2 R* genes, we observed that AvrBs2 activation of a slower Bs2-HR was epistatic to the AvrBs1 activation of a more rapid Bs1-HR (Figure 4). Control strains *Xe* (*avrBs1*) and *Xe* (*avrBs2*) along with control pepper (*Bs1*) and pepper (*Bs2*) were included for comparison to detect the epistatic, slow Bs2-HR at 48 hpi instead of the expected faster Bs1-HR at 18 hpi (Figure 4). The epistasis of the *Xe* activated slower *Bs2* HR over the *Xe* activated faster *Bs1* HR was also confirmed by measuring electrolyte leakage (Supplemental Figure S5A and S5B).

To test whether the Bs2 activation dependent suppression of the AvrBs1/Bs1 fast HR phenotype could be activated in *trans*, we co-inoculated a mixed inoculum of two strains of *Xe* containing either *avrBs1*, *avrBs2* or no effector onto pepper (*Bs1, Bs2*). Again we observed the Bs2 activation dependent suppression of the AvrBs1/Bs1 fast HR phenotype (Supplemental Figure S6A). Control inoculations with single *Xe* effectors, either by individual or mixtures, gave the expected responses on pepper plants with and without the corresponding *R* gene (Supplemental Figure S6). Additionally, the epistasis of the *Xe* activated slower Bs2 HR over the *Xe* activated faster *Bs1* HR was again confirmed by measuring electrolyte leakage (Supplemental Figure S7A).

We hypothesized that this suppression might be accounted for by one of the following: (i) Bs2 activation disrupts *Bs1* activation or (ii) Bs2 activation disrupts TTSS-mediated translocation of AvrBs1 or (iii) *Bs2* activation causes a reduction or loss of induction of AvrBs1. To test the first hypothesis, three *Agrobacterium* strains containing either 35S-*avrBs1,* 35S-*avrBs2* alone or a 35S-*avrBs1*/35S-*avrBs2* tandem construct were inoculated on pepper containing both the *Bs1* and *Bs2 R* genes. If *Bs2* activation disrupts *Bs1* activation, then suppression of AvrBs1/Bs1-dependent HR should occur. However, we did not observe alteration of the fast, Bs1 HR by the slow Bs2 HR activation when both effectors were transiently expressed (Supplemental Figure S8A). The fast Bs1 HR for the co-expressed AvrBs2 and AvrBs1 on pepper (*Bs2*, *Bs1*) was confirmed by measuring electrolyte leakage (Supplemental Figure S8B). In addition, immunoblot analysis detected similar levels of expression for both HA epitope tagged effectors after 24 hours (Supplemental Figure S8C). Therefore, when AvrBs1 and AvrBs2 were simultaneously expressed in plant cells, the Bs2/AvrBs2-dependent HR no longer suppressed the Bs1/AvrBs1*-*dependent HR. This finding is not consistent with the first hypothesis.

**Figure 5:**
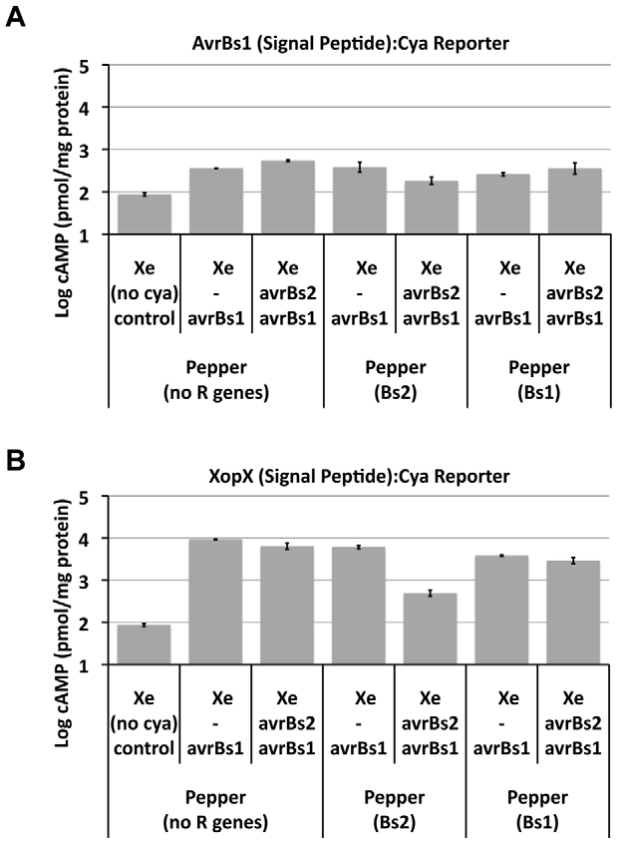
Bs2 activation by AvrBs2 blocks TTSS delivery of two independent effector reporters to host cells. The TTSS effector delivery reporter constructs consisted of the effector promoter and the secretion and translocation signal peptide translationally fused to adenylate cyclase (Cya). These constructs were introduced into *Xe* in tandem with the native effector by single homologous recombination. Pepper (no R genes), pepper (*Bs1*) and pepper (*Bs2*) were sampled 8 hours post-inoculation to avoid in planta multiplication of the reporter strain pairs (with and without *AvrBs2*) and assayed for cyclic AMP (cAMP). TTSS delivered effector-Cya translational fusions into the plant cell and calmodulin from the plant cell leads to elevated levels of cAMP. **A**. AvrBs1_1-212_-Cya reporter in strains *Xe* (*avrBs2* and *avrBs1*) and *Xe* (*avrBs1*) were inoculated into pepper plants (no R genes, *Bs1* or *Bs2*). In planta cAMP levels were assayed. Student t-test was used to compare TTSS delivery of effector reporter in an *Xe* strain with and without *avrBs2* on Pepper (+) *Bs2*; p-values were <0.05. **B**. XopX_1-183_-Cya reporter in strains *Xe* (*avrBs2* and *avrBs1*) and *Xe* (*avrBs1*) were inoculated into pepper plants (no R genes, *Bs1* or *Bs2*). In planta cAMP levels were assayed. Student t-test was used to compare TTSS delivery of effector reporter in an *Xe* strain with and without *avrBs2* on Pepper (+) *Bs2*; p-values were <0.01.

To test our second hypothesis, whether Bs2 activation modulates subsequent *Xe* TTSS effector delivery, the TTSS effector delivery reporter Cya [18] was utilized to quantitatively measure the translocation of two different *Xe* effector-reporters for *avrBs1* and *xopX*. In this assay, the type three secretion and translocation signal peptides for each effector were translationally fused to the reporter Cya. Using homologous recombination, the reporters were marker-exchanged in tandem with the corresponding chromosomal allele of different *Xe* strains so that the wild-type copy of the particular effector was also maintained [18]. Pairs of effector-Cya reporter strains with and without *avrBs2* included the pair of strains *Xe* (*avrBs1*) and *Xe* (*avrBs1*, *avrBs2*) with either AvrBs1_1-212_-Cya reporter (Figure 5A) or XopX_1-183_-Cya reporter (Figure 5B).

Pairs of *Xe* Cya reporter strains, with and without *avrBs2*, were inoculated on pepper (no *R* genes), pepper (*Bs2*) and pepper (*Bs1*). Plants were sampled eight hours post-inoculation to avoid in planta multiplication of the reporter strains [18]. Eight hours post-inoculation is also before visible *R* gene-mediated HR. Because each effector-Cya reporter construct has a unique rate of translocation, each reporter construct was evaluated separately.

When the translocation of AvrBs1 and XopX Cya reporters was assessed in the presence of *Bs2/avrBs2*, the detectable levels of cyclic AMP for both effector-Cya reporters were significantly reduced in comparison to all other combinations where Bs2 was not activated including the *Bs1/AvrBs1* interaction (Figure 5A, 5B).

Additionally, we tested three other pairs of effector-Cya reporter strains with and without *avrBs2* that included the pair of strains *Xe* (*avrBs3*) and *Xe* (*avrBs3*, *avrBs2*) with either AvrBs2_1-212_-Cya reporter, AvrBs3_1-212_-Cya reporter or XopX_1-183_-Cya reporter (Supplemental Figure S9). Again only Bs2 activation was associated with reduced levels of effector-Cya reporter delivery to the host. This is consistent with the hypothesis that the Bs2 activation disrupts general TTSS-mediated translocation of effectors.

To preclude the possibility that Bs2 activation might block calmodulin dependent Cya elevation of *in planta* cyclic AMP levels, we tested *Agrobacterium* transient expression of 35S-AvrBs2:Cya in the presence and absence of *Bs2* at 15 hpi in *N. benthamiana.* Similar elevated levels of cyclic AMP were observed in the presence and absence of Bs2 activation (Supplemental Figure S10A).

Additionally, we evaluated the effect of the GDE catalytic site mutations in AvrBs2 on the TTSS disruption by Bs2 activation with the AvrBs3-Cya reporter *Xe* strain. The set of four effector-Cya reporter *Xe* strains (*avrBs2*, *avrBs2*-E304A/D306A, *avrBs2*-H319A and without *avrBs2*) with the AvrBs3_1-212_-Cya reporter were tested on pepper with or without Bs2. The loss of the GDE catalytic sites in AvrBs2 did not alter the TTSS repression effect of the Bs2/AvrBs2 interaction (Supplemental Figure S10B).

To preclude the possibility that Bs2 activation causes a reduction or loss of induction of TTSS effectors in *Xe*, AvrBs2-Cya, an effector that is also disrupted in delivery to the host by Bs2 activation (Supplemental Figure S9A), was tested for reduction in protein level. Immunoblot assays of high titer inoculation of pepper (w/o *Bs2*) and pepper (*Bs2*) with *Xe* (*avrBs2*), *Xe* (*avrBs2-*Cya), *Xe* (*avrBs2*-E304A/D306A:Cya) and *Xe* (*avrBs2*-H319A:Cya) detected no reductions of protein levels associated with Bs2 activation (Supplemental Figure S10C). Although these results do not support hypothesis (iii) as a broad mechanism targeting all TTSS effectors it does not preclude an AvrBs1 specific targeting for degradation or loss of induction by Bs2 activation. While both 35SAvrBs2:HA and 35S-AvrBs1:HA transiently expressed in pepper were detected in immunoblot analysis we were only able to detect *Xe* expressed AvrBs2:HA but not AvrBs1:HA (data not shown). Low *Xe* expression of AvrBs1 may contribute to the overall low levels of TTSS delivered AvrBs1-Cya reporter compared to all other effector-Cya reporters evaluated. There is also a Bs2 activation specific reduction in the detectable *Xe* delivered AvrBs1-Cya reporter that should correlate with a Bs2 activation specific reduction in the *Xe* delivered AvrBs1. This indirect evidence is all consistent with a Bs2 activation dependent reduction in TTSS delivery of an already lowly expressed AvrBs1 resulting in a lack of the minimal amount of AvrBs1 required to activate a confluent Bs1 HR.

These results led us to conclude that plant cells undergoing a Bs2/AvrBs2 incompatible reaction were able to modulate subsequent effector delivery by the *Xe* TTSS.

## Discussion

### Bacterial GDEs contribute to Xe virulence

Several classes of bacterial TTSS effectors have been characterized based on their enzymatic activities targeting host proteins [6]–[9]. In this study, we identified a GDE domain present in AvrBs2 that is highly conserved in homologs from several species of *Xanthomonas*. In addition to generating a three-dimensional structural model of the GDE domain of AvrBs2 using the crystal structure of a bacterial GDE, we demonstrated that the putative GDE catalytic site of AvrBs2 could functionally replace the catalytic site of the bacterial GDE from *Borrelia hermsii* (*Bh*GlpQ). We further demonstrated that *Xe* strains with mutations in the putative GDE catalytic site of AvrBs2 had reduced bacterial growth in susceptible *bs2* plants, suggesting that glycerolphosphodiesterase activity has an important virulence function in this pathogen. An evolutionary analysis supports this conclusion and demonstrates that the GDE domain in AvrBs2 is under strong purifying selection. Interestingly, the catalytic mutations in GDE did not interfere with the ability of the plant to recognize AvrBs2 through the cognate R protein Bs2 and trigger disease resistance. This finding suggests that recognition of AvrBs2 is independent of its GDE enzyme activity.

Genes with GDE domains have been identified in species across the animal, plant, fungal and bacterial kingdoms [45]–[47]. Although the exact biological functions of most GDE genes are unknown, it has been documented that GDE enzyme activity is directly linked to bacterial pathogenesis in other systems [45]–[47]. For example, in *Borrelia* species, some but not all spirochetes carry GDE genes. It has been demonstrated that spirochetes carrying GDE genes were able to achieve high cell densities (>10^8^/ml) in the blood, whereas spirochetes lacking GDE genes grew too much lower densities (<10^5^/ml) [41], [48]. These results clearly suggest that the GDE gene product could contribute to bacterial virulence, although the exact mechanism is still unclear [40]. Genes similar to GDE have been identified in plants; their products may contribute to plant cell wall biogenesis [49]–[51]. It is possible that bacterial pathogens interfere with the functions of endogenous plant GDEs by either blocking or competing for the same substrates. This hypothesis could be tested in future studies as more information is revealed about plant GDEs and their endogenous substrates.

### The AvrBs2 protein may require a plant cofactor to activate its GDE enzyme activity

In this study, we purified the GST-AvrBs2 fusion protein from *E. coli* and subjected it to a common procedure used to test bacterial proteins for GDE enzyme activity [41]. However, GDE enzyme activity was not detectable using the recombinant GST-AvrBs2. This result could be due to the buffer conditions or the substrates employed, which may not be optimal for AvrBs2 enzyme activity *in vitro*. Interestingly, the *in vitro* GDE enzyme activity of the Arabidopsis putative GDE (AT4G26690) was not confirmed by using a similar testing condition as described in this report [51]. It may suggest that certain plant GDEs prefer different substrates compared to *E. coli* GDE. Our results (Figure 1C and 1D) confirmed that AvrBs2 has a functional GDE catalytic site. However, the amino acid sequences flanking the GDE catalytic site may be important for substrate binding. Since the flanking sequences in AvrBs2 are different from *Bh*GlpQ, AvrBs2 could have a different substrate specificity and not use glycerophosphocoline as substrate.

It is also possible that AvrBs2 requires other plant co-factors to activate its proper folding or its GDE enzyme activity. It is not unusual for a bacterial TTSS effector protein to require plant co-factors for full enzyme activity [1], [6]. For example, the bacterial TTSS effector AvrRpt2 requires plant cyclophilin to activate its protease activity [1], [6]. In this study, however, it was not possible to test whether AvrBs2 required plant cofactors for its GDE enzyme activity by mixing plant total protein extracts because of the high background of endogenous plant GDE activity. By using chimeric proteins, we confirmed that AvrBs2 did possess the functional GDE catalytic site that is essential for GDE enzyme activity. Because the GDE domain is required for the virulence function of AvrBs2, it is possible that AvrBs2 fulfills its virulence function through the GDE-activated hydrolysis of substrates in plant cells. Further investigation to identify the substrates for AvrBs2 enzyme function may help to elucidate the mechanism of the AvrBs2 virulence function and the modulation of *Xe* TTSS.

### The virulence domain of AvrBs2 can be genetically separated from the region that triggers the Bs2-dependent plant immune response

We demonstrated that AvrBs2 carries a GDE domain with catalytic sites required for promoting bacterial virulence. However, GDE activity is not required for the activation of Bs2-dependent disease resistance. Through further genetic analyses, two overlapping AvrBs2 domains were identified: one corresponding to the GDE homologous region and one to a minimal Bs2-activating domain that includes the GDE domain and a C-terminal region. We confirmed that the previously identified mutations in this C-terminal region of AvrBs2 no longer activated Bs2-dependent resistance [24] and several novel mutations were identified that compromised Bs2 activation while having little effect on bacterial virulence. These results show that *Xanthomonas* can overcome *Bs2* resistance without losing the virulence function of AvrBs2. These findings are significant for optimizing the deployment of *Bs2* resistance in field studies because it is important to understand how *Xe* strains can overcome *Bs2* activation but retain the AvrBs2 virulence function. For example, anticipatory breeding could be used to identify new *Bs2* alleles that recognize the AvrBs2 loss-of-recognition mutants (R403P, A410E and Y419A). This scheme would allow us to use molecular breeding to stay ahead of evolving pathogens.

### AvrBs2 activation of Bs2 leads to suppression of subsequent *Xe* TTSS effector delivery to host cells

In this study, we used the AvrBs2/Bs2 system to identify a potentially novel mechanism in plant disease resistance. AvrBs2-dependent activation of Bs2 triggers an unknown plant immunity mechanism, resulting in the suppression or modulation of the TTSS of the bacterial pathogen. In host plants containing the two *R* genes *Bs1* and *Bs2,* we observed epistasis of the *Bs2* activity with a slow, 48-hour HR over the *Bs1* activity with a rapid, 18-hour HR when *avrBs1* and *avrBs2* were present in either a single *Xe* strain or during co-infection into the appropriate pepper plants. A Cya reporter assay demonstrated that this interference was most likely due to the inhibtion of the bacterial TTSS following the AvrBs2/Bs2 interaction. This general inhibition of the subsequent *Xe* TTSS effector-reporter delivery could be detected as early as one hour after inoculation of *Xe* delivering wild-type AvrBs2 to *Bs2* pepper plants.

Recently, it has been reported that the pre-inoculation of non-pathogenic *Pseudomonas fluorescens* or flg21 (a 21-amino-acid peptide from bacterial flagellin) induces PAMP-triggered immunity (PTI) in *Nicotiana tabacum* (tobacco) plants [19]. The PTI subsequently inhibited the HR triggered by the secondary inoculation with *Pseudomonas* carrying TTSS effector genes [19]. Effector-Cya assays confirmed that HR suppression was caused by the restriction of injection of the TTSS effectors into plant cells. From this result, the authors concluded that PTI could directly or indirectly inhibit the injection of TTSS effectors into plant cells [19]. In this report, we demonstrated that the effector-triggered immunity, which was triggered by the interaction of Bs2 and AvrBs2, led to the suppression of the delivery of TTSS effectors into plant cells. It would be interesting to test whether the mechanism of the PTI-based suppression of TTSS is similar to that of the AvrBs2/Bs2 interaction.

Because almost all Gram-negative pathogens, some symbiotic bacteria and several phytopathogenic bacteria have similar TTSS machineries [52]–[54], it is possible that the conserved components of the TTSS machinery also serve as PAMPs that are specifically recognized by plant extra- or intracellular receptors, triggering plant immunity [55]. It would be intriguing to test the hypothesis that the interaction of AvrBs2 with Bs2 directly or indirectly modifies the plant cell walls, subsequently blocking the penetration of the TTSS pilus across the plant cell walls. It would also be interesting to explore whether the TTSS suppression triggered by AvrBs2/Bs2 is common in other R protein/effector interactions in other plant species. Answering these questions may reveal whether plants employ TTSS suppression as a general immune response to help inhibit the growth of invasive bacterial pathogens.

## Materials and Methods

### Strains and growth

*Escherichia coli* strains DH5α, Top10, BL21(DE3) and DB3.1 as well as *Agrobacterium tumefaciens* strain C58C1 were grown on Luria-Bertani agar containing the appropriate antibiotics at 37°C (for *E. coli*) and 28°C (for *A. tumefaciens*). *Xanthomonas* strains were grown on nutrient yeast glucose agar [56] containing the appropriate antibiotics at 28°C. The *Xanthomonas* strains used were GM98-38 *Xe* (*avrBs3*), GM98-38-1 *Xe* (*avrBs2*, *avrBs3*) [24], 85–10 *Xe* (*avrBs2*, *avrBs1*) [31] and 69–1 *Xe* (*avrBs2*) [25] . Various constructs in *E.* coli were transferred to *Xanthomonas* and *A. tumefaciens* C58C1 by tri-parental mating with DH5α (RK600) acting as helper strain [57].

Electrolyte leakage of 1.5 cm^2^ pepper leaf disc post inoculation with *Xe* strains at 2×10^8^ CFU/ml and rocked gently in 4 ml water for 1 hour. Conductance was measured with an Thermo Orion conductance meter (model 105A+) in microSiemens/cm (uS).

*Nicotiana benthamiana*, tomato cv. VF36, *Bs2* transgenic *Nicotiana benthamiana* and VF36 and pepper lines ECW-0 (no R gene control), ECW-20R (*Bs2*), ECW-10R (*Bs1*) and ECW-123R (*Bs1*, *Bs2* and *Bs3*) were grown in the greenhouse before and after inoculation at 24°C under 16 hours light/8 hours dark cycles.

### Homology-based modeling

The MODELLER software package [37] was used to create a comparative protein structural model for AvrBs2 using the solved crystal structure of 1o1z A as a template. The Chimera package was used to perform structural alignments and generate molecular graphics images [58].

### *avrBs2* GDE catalytic site subcloning and protein purification

The full-length *avrBs2* gene was amplified as a *Bam*HI-*Sal*I fragment by using the following primer set: 5′-caccGGATCCATGCGTATCGGTCCTCTGCAACCTTC-3′ and 5′-GTCGACATCCGTCTCCGTCTGCCTGGCCT-3′. The resulting PCR fragment was cloned into the same sites of the protein expression vector pGEX4T-1 (GE Healthcare, NJ). The GDE positive control gene *Borrelia hermsii BhGlpQ* was amplified from a plasmid provided by Dr. Tom Schwan (University of Montana, Missoula, MT, USA) by using the following primer set: 5′-caccGGATCCTGTCAGGGCGAAAAAATGAGTCA-3′ and 5′-GTCGAC TGGTTTTATTTTTGTGATGAA-3′. The PCR product was cloned into the *Bam*HI/*Sal*I sites of pGEX4T-1 (GE Healthcare, Piscataway, NJ). An overlap extension PCR method was applied to generate the chimeric genes *BhGlpQ-avrBs2*-wt and *BhGlpQ-avrBs2*-E304A/D306A. The catalytic domain of wild-type avrBs2 was first amplified with the following primer set: 5′-caccGGATCCTGTCAGGGCGAAAAAATGAGTCA-3′ and 5′-GCACGCCATCGGAACTGACTTCGACGTCCAGCTCTAGGTAGTCAGCTCCTAAGGCAT-3′. The catalytic domain of avrBs2-E304A/D306A was amplified with the following primer set: 5′-caccGGATCCTGTCAGGGCGAAAAAATGAGTCA-3′ and 5′-GCACGCCATCGGAACTGACTTCGACGGCCAGCGCTAGGTAGTCAGCTCCTAAGGCAT-3′. The derived PCR products were used as templates for another round of amplification with the following primer set: 5′-caccGGATCCTGTCAGGGCGAAAAAATGAGTCA-3′ and5′-GTTTGTTGTTGTATCAAGTTCTGGATCGTGCATCAACACCGGCACGCCATCGGAACTGA-3′. The resulting product was the N-terminal chimera with *BhglpQ* genes carrying the GDE catalytic domain from either the wild-type or the mutant *avrBs2* gene.

The other portion of the DNA sequence of the *BhGlpQ* gene was amplified with the following primer set: 5′-TCAGTTCCGATGGCGTGCCGGTGTTGATGCACGATCCAGAACTTGATACAACAACAAAC-3′ and 5′-GTCGACTGGTTTTATTTTTGTGATGAA-3′. The resulting two portions of the chimeric *BhglpQ* gene were re-amplified with the following primer set: 5′-caccGGATCCTGTCAGGGCGAAAAAATGAGTCA-3′ and 5′-GTCGAC TGGTTTTATTTTTGTGATGAA-3′. The PCR products were purified by a gel-purification kit (Bioneer, CA) and cloned into the *Bam*HI/*Sal*I sites of pGEX4T-1 (GE Healthcare, NJ). The DNA sequences of all clones were confirmed by sequencing.

The protein expression constructs were transformed into *E. coli* strain BL21(DE3) by electroporation and were grown in liquid LB medium supplemented with 50 µg/ml ampicillin at 28°C/220 rpm to OD_600_ = 0.4; 0.5 mM IPTG was added to the culture for 6 hours to induce protein expression. The cells were harvested and disrupted by sonication in cold PBS buffer (147 mM NaCl, 2.7 mM KCl, 10 mM Na_2_HPO_4_, 2 mM KH_2_PO_4_, pH = 7.4) supplemented with 1% Triton X-100. The cell debris was cleared by centrifugation at 12,000 g for 20 min. The soluble GST fusion proteins were purified using Glutathione Sepharose following the protocol provided by the manufacturer (GenScript USA Inc., NJ, USA). The fusion proteins were eluted in 50 mM Tris-Cl, pH = 8.0, supplemented with 10 mM reduced glutathione. All protein samples were stored on ice before the enzyme assays.

### GDE enzyme assays

The enzyme activity of the purified GST-fusion proteins was determined using an enzyme-coupled spectrophotometric assay to measure the amount of G3P that was released by the glycerophosphodiester phosphodiesterase reaction. The reaction mixture contained 0.2 M hydrazine-glycine buffer, pH = 9.0, 0.5 mM NAD, 10 U/ml G3P dehydrogenase (Sigma G6880), 10 mM CaCl_2_, 0.5 mM Sn-glycerol-3-phosphocholine (G5291), and the GST-fusion proteins at several pre-set concentrations. The reaction mixture was incubated at 30°C in a 96-well plate for 1 h until the oxidation of G3P by G3P dehydrogenase was complete. The G3P concentration was determined from the absorbance change at 340 nm by using the BioTek plate reader (BioTek Instruments, Inc., VT, USA).

### Site-directed mutagenesis of *avrBs2* in the *Xe* genome and construction of Cya fusions for TTSS effector delivery reporters

Mutants formed by homologous recombination of the genomic copy of *avrBs2* in *Xe* were constructed as previously described [18], [59]. The *avrBs2* open reading frame was first PCR amplified with a *Sal*I site at the 5′-end and a *Bam*HI site at the 3′-end and cloned directionally into pBluescript KS+. This intermediate construct was mutagenized using the QuikChange Site-Directed Mutagenesis kit (Stratagene, CA) to incorporate the two GDE catalytic site mutations (E304A/D306A, H319A and Y419A) using overlapping forward and reverse primers for the E304A/D306A sequence (5′-CAATCTGGCGCTGGCCGTCGAAG-3′), H319A sequence (5′- GTGTTGATGGCCGATTTCAG-3′) and for the Y419A sequence (5′- GCCAAGTACGCCACGGGCGG-3′). The resultant mutant constructs were digested with *Not*1 and *Bam*H1, and T4 DNA polymerase was used to create blunt ends. The blunt-ended fragments were then cloned into the suicide vector pLVC18L, which has a col E1 replicon and contains the highly efficient mob region from pRSF1010 [18], cut with *Xba*I and *Sma*I, and filled using T4 DNA polymerase to make pLVC18*avrBs2* (E304A/D306A, H319A and Y419A). The three constructs were then mobilized into *Xe* (*avrBs2*, *avrBs3*) and rescued by tetracycline selection of a single recombination event into the genomic copy of *avrBs2*. Second-site resolution crossover events were identified as tetracycline-sensitive single colonies from cultures grown in the absence of tetracycline. PCR amplification and sequencing were used to confirm a double homologous recombination event for either the E304A/D306A, H319A or Y419A. All bacterial growth assays in pepper and tomato were performed as previously described [25].

Two mutant strains *Xe* (E304A/D306A and H319A) were further modified by homologous recombination to add Cya as a C-terminal translational fusion as previously reported [18].

Double homologous genomic recombination was used to delete the *avrBs2* locus in strains 85–10 *Xe* (*avrBs2*, *avrBs1*) and 69–1 *Xe* (*avrBs2*) to make *Xe* (*avrBs1*) and *Xe* (no effector) respectively using p815:*avrBa2*:GM as previously described [23].

### Epitope-tagged AvrBs2 deletions and mutations and *Agrobacterium-*mediated transient expression

All *avrBs2* deletions and mutations were first cloned into pENTR/D-TOPO (Invitrogen) as previously described [59]. Each construct began with a start codon and ended without a stop codon so that the HA epitope and stop codon of the destination vector would be maintained after transfer. For *Agrobacterium-*mediated transient expression from the 35S promoter and C-terminal HA epitope tagging, pMD1 was first digested with *Xho*1. The HA epitope and the stop codon linker (5′- CTCGAGTATCCCTACGACGTACCAGACTACGCATAGCTCGAG-3′) were cloned in and then re-opened at the *Sma1* site, and the ccdB cassette A (Invitrogen) was cloned in to create the destination vector pMD1-Des-HA. All pENTR-*avrBs2* constructs were then transferred to pMD1-Des-HA using LR clonase (Invitrogen).

For AvrBs1:HA and AvrBs2:HA *Agrobacterium-*mediated transient expression constructs both full length effectors were cloned into pENTR/D-TOPO with N-terminal *Xba*I site and a Cterminal HA epitope tag (5′- GGATCCTACCCATACGATGTTCCTGACTATGCGGGCTATCCCTATGACGTCCCGGACTATGCAGGATAGGAGCTC-3′) followed by a *Sac*I site. These were then subcloned into pMD1. The pMD1-AvrBs2:HA construct was further modified by re-opening at the single *Bsa*I site and the ccdB cassette B (Invitrogen) cloned in to create a destination vector. The *Hind*III-*Eco*RI 35S-nosTerminator fragment was cloned into pENTR/D TOPO and then the AvrBs1:HA *Xba*I-*Sac*I fragment was subcloned in. This pENTR-35S-AvrBs1:HA was transferred into the pMD1-AvrBs2:HA destination vector using LR clonase (Invitrogen) to create a double effector binary vector for *Agrobacterium* transient expression.

The binary deletion and mutation constructs were transferred to *Agrobacterium* (C58C1) for transient expression in *Nicotiana benthamiana* and pepper, as previously described [25].

Immunoblot analysis protocol was previously described [26].

### Generating the Cya reporter fusion with the *Xe* effectors *avrBs1, avrBs2, avrBs3* and *XopX*

Two effector-Cya reporters from *avrBs1* and *avrBs3* were made by directional cloning PCR products into Gateway-compatible pENTR/D-TOPO (Invitrogen) and then translationally fused to Cya by LR clonase (Invitrogen) into the suicide destination vector pDDesCya [59]. The effector PCR products of 1352 base pair for *avrBs1* and 950 bp for *avrBs3* included the promoter region and the first 212 codons of AvrBs1 and the first 107 codons of AvrBs3 were used to create AvrBs1_1-212_-Cya and AvrBs3_1-107_-Cya, respectively. The two previously constructed pDDesCya effector-Cya reporters for AvrBs2_1-98_-Cya and XopX_1-183_-Cya, along with AvrBs3_1-107_-Cya and AvrBs1_1-212_-Cya, were introduced into *Xe* by genomic single recombination rescues of these constructs. This recombination still maintained the wild-type genomic copy of the particular effector [18]. The pairs of effector-Cya reporter strains with and without *avrBs2* included the three-strain pairs of *Xe* (*avrBs3*) and *Xe* (*avrBs3*, *avrBs2*) with either reporter AvrBs2_1-98_-Cya, AvrBs3_1-107_-Cya or XopX_1-183_-Cya. Also included were the two-strain pairs of *Xe* (*avrBs1*) and *Xe* (*avrBs1*, *avrBs2*) with either XopX_1-183_-Cya or AvrBs1_1-212_-Cya. Additionally the pDDesCya with AvrBs3_1-107_-Cya was introduced into strains *Xe* (*avrBs2*-E304A/D306A or H319A) by genomic single recombination rescues of these constructs.

The Cya was added to the C-terminus of *Xe* catalytic mutants of AvrBs2 as previously described [18].

The 35S- *avrBs2*:Cya construct was made by replacing the *Bam*HI-*Sac*I GFP fragment from pMD1- *avrBs2*:GFP [18]. with a *Bam*HI-*Sac*I Cya fragment. This construct was introduced into *Agrobacterium* for transient expression as previously described [26].

Plant cyclic AMP (cAMP) levels eight hours post-inoculation were measured as previously described [18]. Sampling at eight hours post-inoculation will avoid in planta multiplication of the reporter strains. Eight hours post-inoculation is also long before the development of any R gene-mediated HR.

## Supporting Information

Supplemental Figure S1 **Inoculation of near-isogenic pepper (** ***Bs2*** **) and pepper (w/o** ***Bs2*** **) with high-density suspensions of** ***Xe*** **(2×10^8^ CFU/ml).** Bs2-dependent brown necrotic HR detected at 96 hours post-inoculation for *Xe* (*avrBs2*) strain GM98-38-1 and double homologous recombination mutants for the putative GDE catalytic site *Xe* (*avrBs2*-E304A/D306A) and Xe (avrBs2-H319A). Also the AvrBs2 mutations identified in *Xe* strains isolated from bacterial spot diseased pepper (*Bs2*) were recombined into GM98-38-1 for *Xe* (*avrBs2*-R403P) produced no HR, similar to the control *X*e (w/o *avrBs2*). The other mutant *Xe* (*avrBs2*-A410E) produced intermediate HR with light brown necrosis detected. In pepper (w/o *Bs2*), only the strains w/o *avrBs2* or with putative catalytic site mutations gave an altered high-density virulent phenotype.
(TIF)

Supplemental Figure S2 **Confirm** ***Xe*** **strains with GDE catalytic site mutations are not altered in TTSS delivery to host and are not altered in Bs2 activated HR. A.** The TTSS effector reporter adenylate cyclase (Cya) was translationally fused on the C terminus of the gemonic copy of AvrBs2 and the AvrBs2 catalytic site mutants. These *Xe* TTSS reporter inoculations on pepper were sampled 8 hours post-inoculation to avoid *in planta* multiplication *Xe* and in planta cyclic AMP (cAMP) levels were assayed. No alteration of AvrBs2 delivery for catalytic site mutations. **B.** Inoculation of pepper (*Bs2*) and pepper (w/o *Bs2*) with high-density suspensions of *Xe* (2×10^8^ CFU/ml) at 48 hpi. No alteration in HR phenotype for *Xe* strains with Cya translational reporters.
(TIF)

Supplemental Figure S3 **Confirmation of protein expression for** ***Agrobacterium*** **transient constructs for minimum domain and key amino acids mutations of AvrBs2 required for Bs2 activation. A**. Immunoblot analysis with anti-HA showing *Agrobacterium*-mediated transient protein expression of HA epitope-tagged constructs of the various *avrBs2* deletions and mutations of the minimal Bs2-HR activation domain (∼31 kDa). Ponceau S staining of immunoblot as loading control. **B.** Near-isogenic pepper with and without Bs2 inoculated for *Agrobacterium* transient expression (48 hpi at 2×10^8^ CFU/ml) with the 35S-HA epitope tagged constructs of the various AvrBs2 deletions and mutations of the minimal Bs2-HR activation domain.
(TIF)

Supplemental Figure S4 **Evaluation of loss of Bs2 activation mutant AvrBs2 (Y419A) in** ***Xe.*** **A.** In planta pathogen growth assay for *Xanthomonas* strains GM98-38 *X*e (w/o *avrBs2*), GM 98-38-1 *Xe* (*avrBs2*), and GM98-38-1 *Xe* (Y419A) exchange mutant. Host plants pepper (w/o *Bs2*) and pepper (*Bs2*). Exchange mutant *Xe* (Y419A) was unable to completely overcome Bs2 resistance. **B. **Inoculation of near-isogenic pepper (*Bs2*) and pepper (w/o *Bs2*) with high-density suspensions of *Xe* (2×10^8^ CFU/ml). Exchange mutant *Xe* (Y419A) gave a light brown necrotic HR on pepper (*Bs2*).
(TIF)

Supplemental Figure S5 **Electrolyte leakage to confirm the slower Bs2-HR (48 hpi) is epistatic to the faster Bs1-HR (18 hpi) for high-density (1.5×10^8^ CFU/ml)** ***Xe*** **inoculations of pepper lines with both R genes (** ***Bs2*** **and** ***Bs1*** **). A**. At 18 hpi electrolyte leakage of inoculated leaf disc in water were measured with a conductance meter in microSiemens/cm (uS). High levels of electrolytes correlated with the corresponding HR phenotypes reported in figure 4. **B.** At 26 hpi electrolyte leakage of inoculated leaf disc in water were measured with a conductance meter in microSiemens/cm (uS). High levels of electrolytes also correlated with the corresponding HR phenotypes reported in figure 4.
(TIF)

Supplemental Figure S6 **The slower Bs2-HR (48 hpi) from mixed high-density (1.5×10^8^ CFU/ml) inoculation of independent** ***Xe*** **strains one with AvrBs1 and one with AvrBs2 was epistatic to the faster Bs1-HR (18 hpi) for pepper (** ***Bs1, Bs2*** **). A.** Near-isogenic pepper lines with bacterial spot resistance genes (*Bs1, Bs2* and the combination of *Bs1* and *Bs2*), at 18 and 48 hours post-inoculation (hpi) with the mixed strains *Xe* (*avrBs1*) and Xe (*avrBs2*). When the mixed strains *Xe* (*avrBs1*) and Xe (*avrBs2*) were co inoculated on pepper (*Bs1*, *Bs2*) the fast Bs1/AvrBs1 HR was again not detected at 18 hpi. **B.** All pepper (no R-gene) control inoculations with single *Xe* effector, either by individual or mixtures, gave the expected responses.
(TIF)

Supplemental Figure S7 **Electrolyte leakage to confirm slower Bs2-HR (48 hpi) from mixed high-density (1.5×10^8^ CFU/ml) inoculation of independent** ***Xe*** **strains one with AvrBs1 and one with AvrBs2 was epistatic to the faster Bs1-HR (18 hpi) for pepper (** ***Bs1, Bs2*** **).** At 18 hpi electrolyte leakage of inoculated leaf disc in water were measured with a conductance meter in microSiemens/cm (uS). High levels of electrolytes correlated with the corresponding HR phenotypes reported in Supplemental figure 6A.
(TIF)

Supplemental Figure S8 **Bs2 activation dependent suppression of the AvrBs1/Bs1 fast HR phenotype not observed when expressed inside plant cells via** ***Agrobacterium*** **transient expression. A.** *Agrobacterium* transient expression strains containing 1. *Agro* 35S-*avrBs1,* 2. *Agro* 35S-*avrBs1* + 35S-*avrBs2* and 3. *Agro* 35S-AvrBs2 were inoculated on pepper plants containing the *Bs1,* (*Bs1* and *Bs2*) and no resistance genes. During the co-expression of AvrBs2 and AvrBs1 no epistasis was observed as the phenotype of the Bs1 HR was not altered by the co-expression of AvrBs2. **B.** Electrolyte leakage was observed for the same combinations as shown in panel A. The electrolyte leakage phenotype of the Bs1 HR was observed when both AvrBs2 and AvrBs1 were co-expressed in *Agro* confirming that there was no epistasis when the genes are co-expressed *in planta.* **C.** Immunoblot detection of AvrBs2-HA and AvrBs1-HA expressed in pepper plants via *Agrobacterium* transient expression at 0 and 24 hpi. This result showed that the activation of Bs2-specified resistance did not interfere with the detection of the Bs1 protein.
(TIF)

Supplemental Figure S9 **Bs2 activation by AvrBs2 blocked subsequent TTSS delivery of multiple effectors to host cells.** TTSS effector delivery reporter constructs consisted of the effector promoter and the secretion and translocation signal peptides translationally fused to adenylate cyclase (Cya). Pepper plants (no R genes, *Bs2* or *Bs3*) were sampled 8 hours post-inoculation to avoid in planta multiplication of the reporter strain pairs (with and without *avrBs2*) and assayed for cyclic AMP (cAMP). Effector-Cya translational fusion and calmodulin from the plant cell led to elevated levels of cAMP. **A.** AvrBs2_1-212_-Cya reporter in *Xanthomonas* strains GM98-38-1 *Xe* (*avrBs2* and *avrBs3*) and *Xe* (*avrBs3*) inoculated onto pepper plants (no R genes, *Bs3* or *Bs2*). In planta cAMP levels were assayed. Student t-test was used to compare TTSS delivery of effector reporter in an *Xe* strain with and without *avrBs2* on *Bs2* pepper plants; p-values were <0.01. **B.** AvrBs3_1-212_-Cya reporter in *Xanthomonas* strains GM98-38-1 *Xe* (*avrBs2* and *avrBs3*) and *Xe* (*avrBs3*) inoculated onto pepper plants (no R genes, *Bs3* or *Bs2*). In planta cAMP levels were assayed. Student t-test was used to compare TTSS delivery of effector reporter in an *Xe* strain with and without *avrBs2* on *Bs2* pepper plants; p-values were <0.01. **C.** XopX_1-183_-Cya reporter in *Xanthomonas* strains GM98-38-1 *Xe* (*avrBs2* and *avrBs3*) and *Xe* (*avrBs3*) inoculated onto pepper plants (no R genes, *Bs3* or *Bs2*). In planta cAMP levels were assayed. Student t-test was used to compare TTSS delivery of effector reporter in an *Xe* strain with and without *avrBs2* on *Bs2* pepper plants; p-values were <0.01.
(TIF)

Supplemental Figure S10 **Elevated levels of** ***in-planta*** **cyclic AMP resulting from** ***Agrobacterium*** **transient expression of 35S-** ***avrBs2*** **:Cya was not blocked by Bs2 activation. Also** ***avrBs2*** **GDE catalytic mutations were still able to block subsequent TTSS effector delivery to pepper host. Also Bs2 activation does not change effector protein levels in** ***Xe.*** **A.** *Agrobacterium* transient expression of 35S-*avrBs2*:Cya in *Nicotiana benthamiana* with and without *Bs2* sampled at 15 hpi were similar for elevated levels of cyclic AMP. **B.** AvrBs3 (signal peptide):Cya TTSS effector reporter recombined into *Xanthomonas* strains GM98-38-1 *Xe* (*avrBs2*, *avrBs2*-E304A/D306A or *avrBs2-*H319A) had similar reduced levels of cyclic AMP in the presence of *Bs2* compared to pepper host without *Bs2*. **C.** Immunoblot assays of high titer inoculation (5×10^8^ CFU/ml) of pepper (w/o *Bs2*) and pepper (*Bs2*) at 8 hpi with *Xe* (*avrBs2*), *Xe* (*avrBs2-*Cya), *Xe* (*avrBs2*-E304A/D306A:Cya) and *Xe* (*avrBs2*-H319A:Cya) detected no reductions of protein levels associated with Bs2 activation.
(TIF)
