# Supplementary figures and images for: Computational and Biochemical Analysis of the Xanthomonas Effector AvrBs2 and Its Role in the Modulation of Xanthomonas Type Three Effector Delivery
Source: PLoS Pathog. 2011 Dec 1;7(12):e1002408. doi: 10.1371/journal.ppat.1002408 (PMC3228805; doi:10.1371/journal.ppat.1002408)

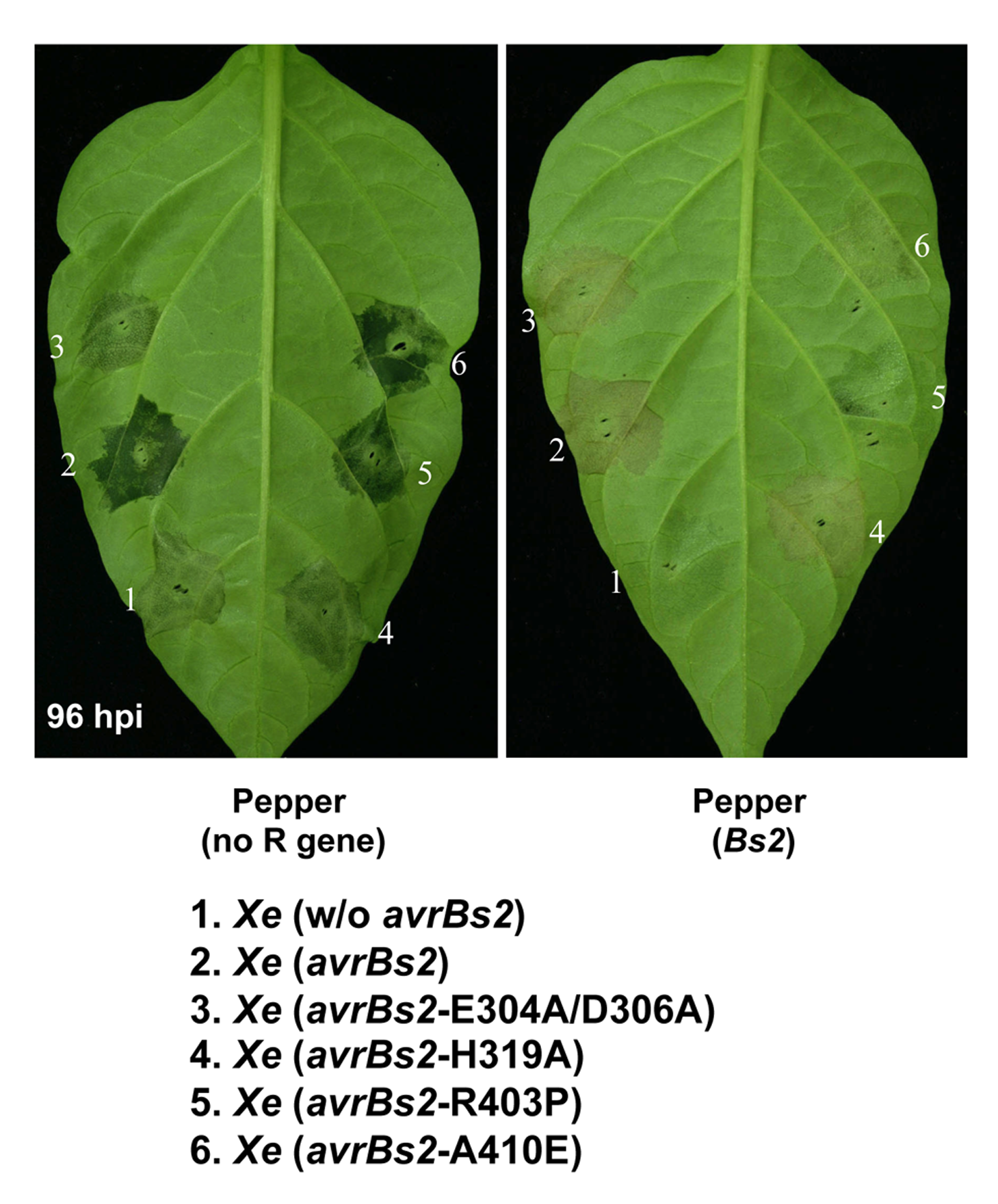

Supplement: Supplemental Figure S1 — Inoculation of near-isogenic pepper ( Bs2 ) and pepper (w/o Bs2 ) with high-density suspensions of Xe (2×10^8^ CFU/ml). Bs2-dependent brown necrotic HR detected at 96 hours post-inoculation for Xe (avrBs2) strain GM98-38-1 and double homologous recombination mutants for the putative GDE catalytic site Xe (avrBs2-E304A/D306A) and Xe (avrBs2-H319A). Also the AvrBs2 mutations identified in Xe strains isolated from bacterial spot diseased pepper (Bs2) were recombined into GM98-38-1 for Xe (avrBs2-R403P) produced no HR, similar to the control Xe (w/o avrBs2). The other mutant Xe (avrBs2-A410E) produced intermediate HR with light brown necrosis detected. In pepper (w/o Bs2), only the strains w/o avrBs2 or with putative catalytic site mutations gave an altered high-density virulent phenotype. (TIF) [file ppat.1002408.s001.tif]

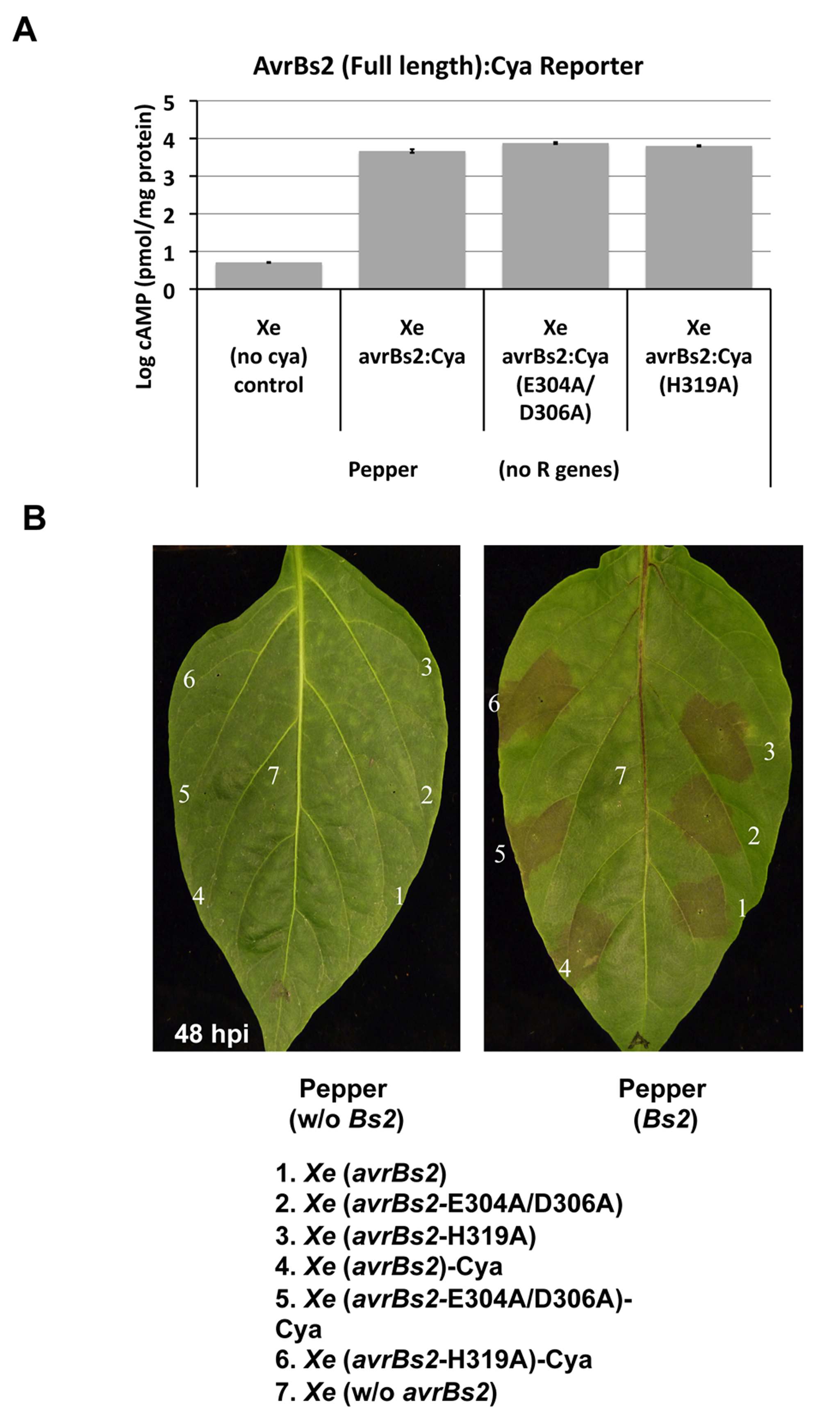

Supplement: Supplemental Figure S2 — Confirm Xe strains with GDE catalytic site mutations are not altered in TTSS delivery to host and are not altered in Bs2 activated HR. A. The TTSS effector reporter adenylate cyclase (Cya) was translationally fused on the C terminus of the gemonic copy of AvrBs2 and the AvrBs2 catalytic site mutants. These Xe TTSS reporter inoculations on pepper were sampled 8 hours post-inoculation to avoid in planta multiplication Xe and in planta cyclic AMP (cAMP) levels were assayed. No alteration of AvrBs2 delivery for catalytic site mutations. B. Inoculation of pepper (Bs2) and pepper (w/o Bs2) with high-density suspensions of Xe (2×10^8^ CFU/ml) at 48 hpi. No alteration in HR phenotype for Xe strains with Cya translational reporters. (TIF) [file ppat.1002408.s002.tif]

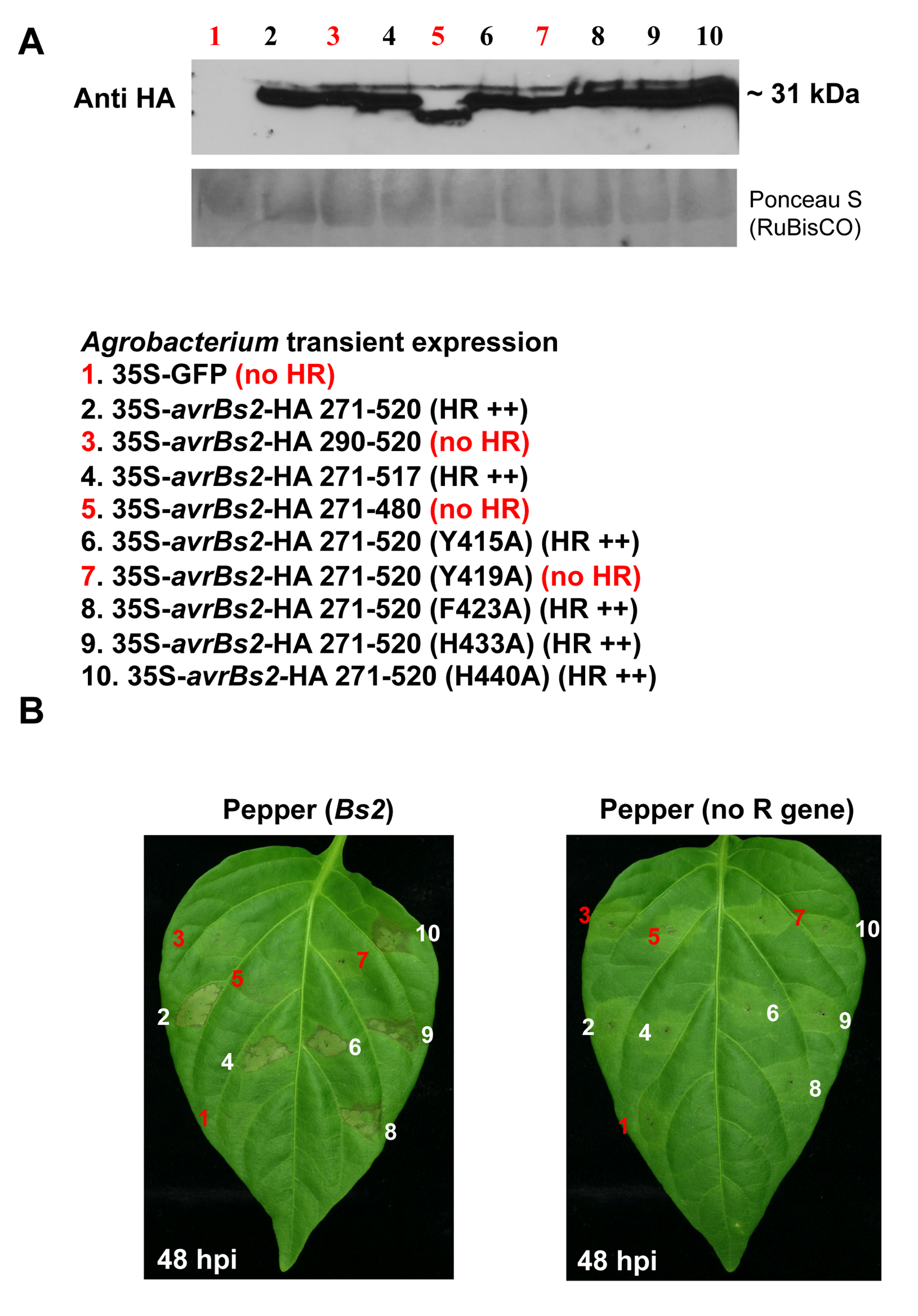

Supplement: Supplemental Figure S3 — Confirmation of protein expression for Agrobacterium transient constructs for minimum domain and key amino acids mutations of AvrBs2 required for Bs2 activation. A. Immunoblot analysis with anti-HA showing Agrobacterium-mediated transient protein expression of HA epitope-tagged constructs of the various avrBs2 deletions and mutations of the minimal Bs2-HR activation domain (∼31 kDa). Ponceau S staining of immunoblot as loading control. B. Near-isogenic pepper with and without Bs2 inoculated for Agrobacterium transient expression (48 hpi at 2×10^8^ CFU/ml) with the 35S-HA epitope tagged constructs of the various AvrBs2 deletions and mutations of the minimal Bs2-HR activation domain. (TIF) [file ppat.1002408.s003.tif]

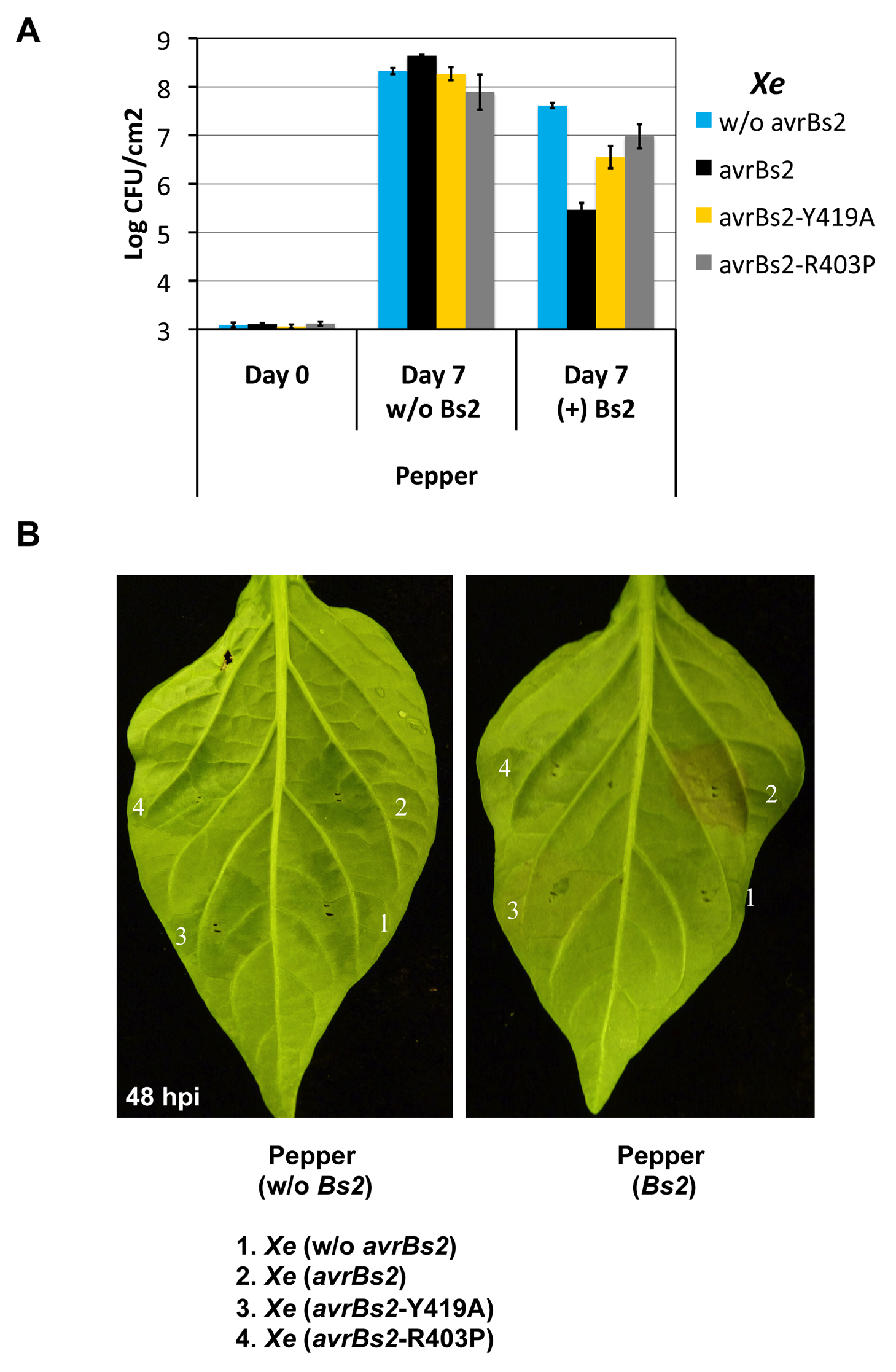

Supplement: Supplemental Figure S4 — Evaluation of loss of Bs2 activation mutant AvrBs2 (Y419A) in Xe. A. In planta pathogen growth assay for Xanthomonas strains GM98-38 Xe (w/o avrBs2), GM 98-38-1 Xe (avrBs2), and GM98-38-1 Xe (Y419A) exchange mutant. Host plants pepper (w/o Bs2) and pepper (Bs2). Exchange mutant Xe (Y419A) was unable to completely overcome Bs2 resistance. B. Inoculation of near-isogenic pepper (Bs2) and pepper (w/o Bs2) with high-density suspensions of Xe (2×10^8^ CFU/ml). Exchange mutant Xe (Y419A) gave a light brown necrotic HR on pepper (Bs2). (TIF) [file ppat.1002408.s004.tif]

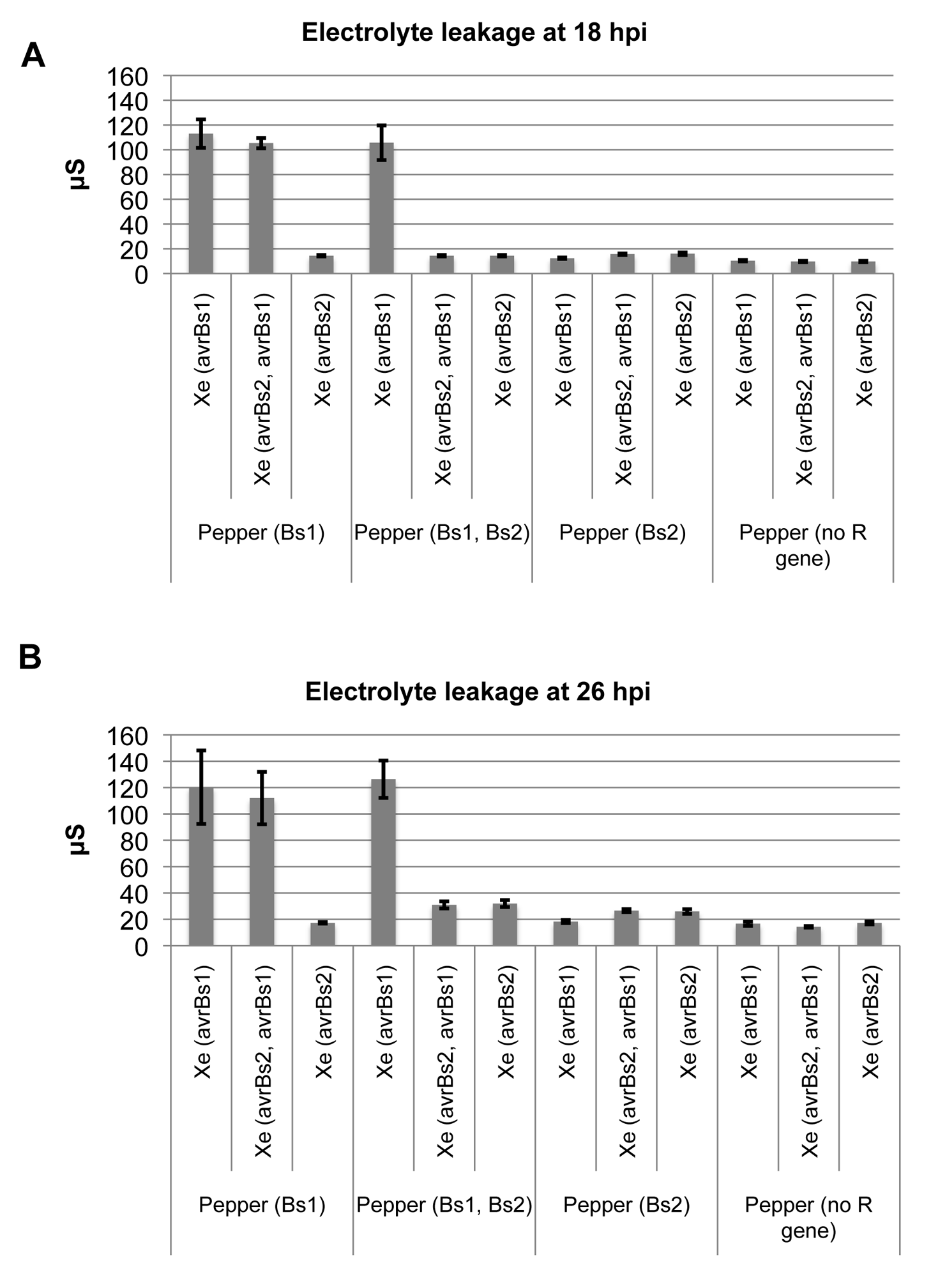

Supplement: Supplemental Figure S5 — Electrolyte leakage to confirm the slower Bs2-HR (48 hpi) is epistatic to the faster Bs1-HR (18 hpi) for high-density (1.5×10^8^ CFU/ml) Xe inoculations of pepper lines with both R genes ( Bs2 and Bs1 ). A. At 18 hpi electrolyte leakage of inoculated leaf disc in water were measured with a conductance meter in microSiemens/cm (uS). High levels of electrolytes correlated with the corresponding HR phenotypes reported in figure 4. B. At 26 hpi electrolyte leakage of inoculated leaf disc in water were measured with a conductance meter in microSiemens/cm (uS). High levels of electrolytes also correlated with the corresponding HR phenotypes reported in figure 4. (TIF) [file ppat.1002408.s005.tif]

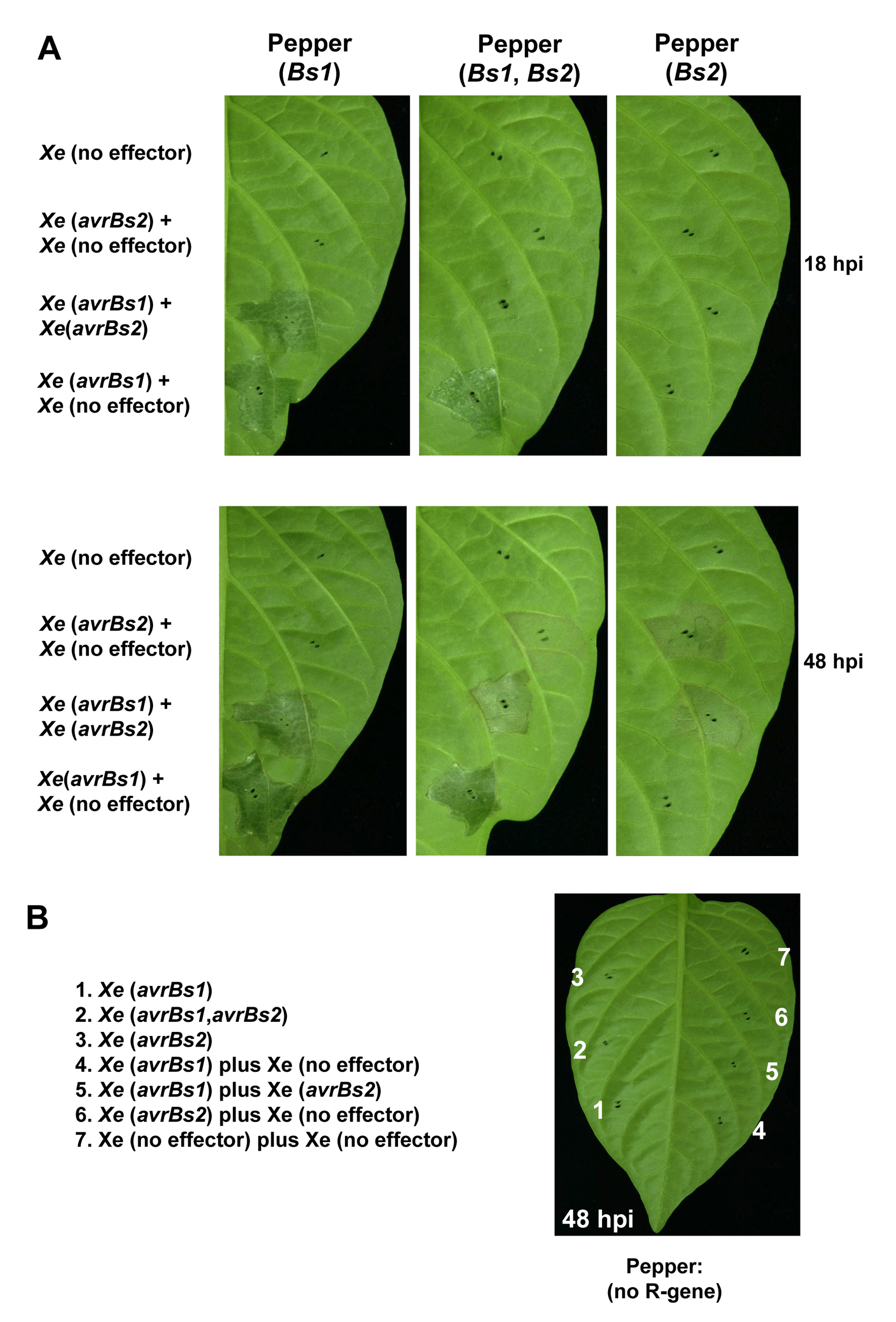

Supplement: Supplemental Figure S6 — The slower Bs2-HR (48 hpi) from mixed high-density (1.5×10^8^ CFU/ml) inoculation of independent Xe strains one with AvrBs1 and one with AvrBs2 was epistatic to the faster Bs1-HR (18 hpi) for pepper ( Bs1, Bs2 ). A. Near-isogenic pepper lines with bacterial spot resistance genes (Bs1, Bs2 and the combination of Bs1 and Bs2), at 18 and 48 hours post-inoculation (hpi) with the mixed strains Xe (avrBs1) and Xe (avrBs2). When the mixed strains Xe (avrBs1) and Xe (avrBs2) were co inoculated on pepper (Bs1, Bs2) the fast Bs1/AvrBs1 HR was again not detected at 18 hpi. B. All pepper (no R-gene) control inoculations with single Xe effector, either by individual or mixtures, gave the expected responses. (TIF) [file ppat.1002408.s006.tif]

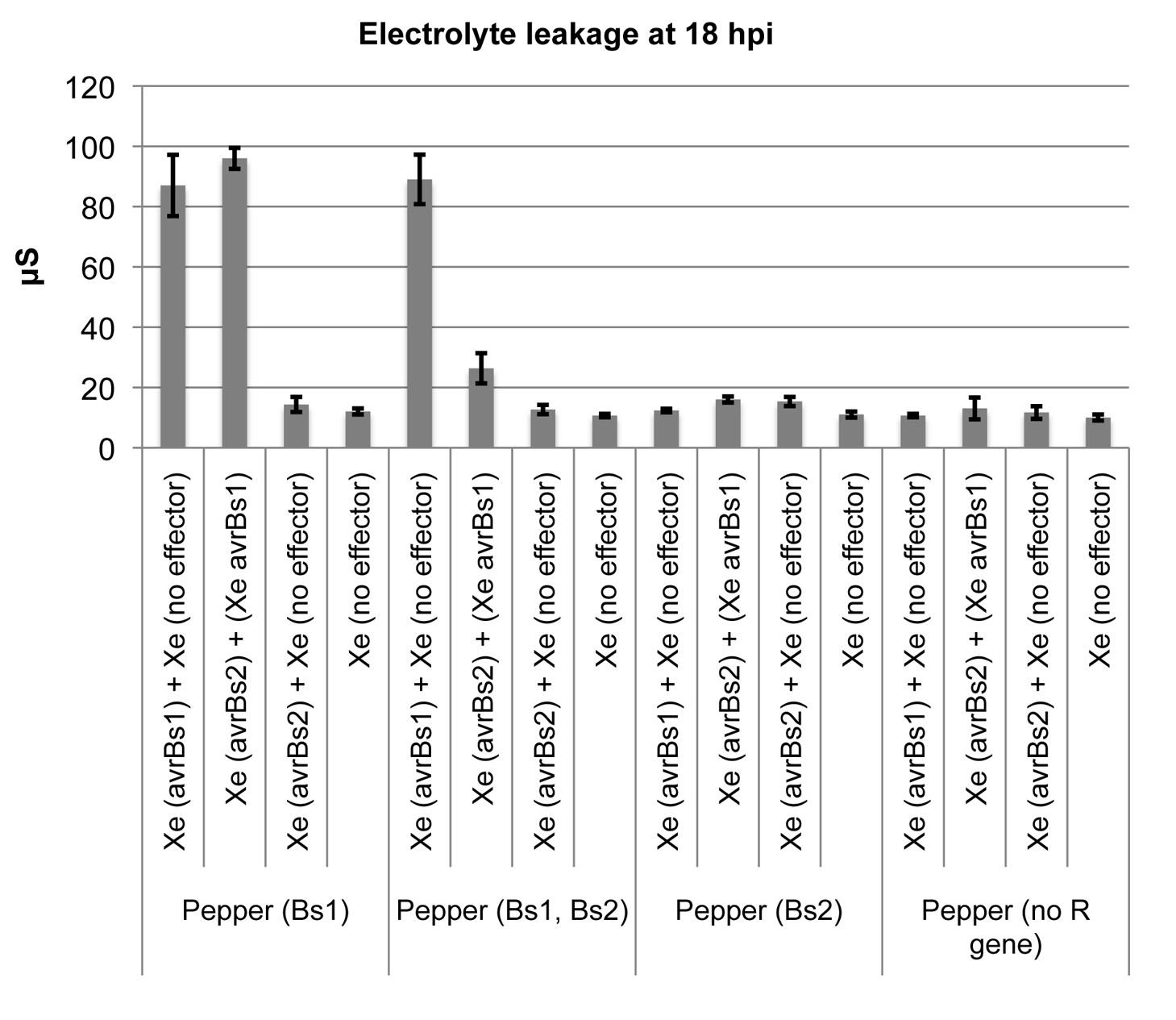

Supplement: Supplemental Figure S7 — Electrolyte leakage to confirm slower Bs2-HR (48 hpi) from mixed high-density (1.5×10^8^ CFU/ml) inoculation of independent Xe strains one with AvrBs1 and one with AvrBs2 was epistatic to the faster Bs1-HR (18 hpi) for pepper ( Bs1, Bs2 ). At 18 hpi electrolyte leakage of inoculated leaf disc in water were measured with a conductance meter in microSiemens/cm (uS). High levels of electrolytes correlated with the corresponding HR phenotypes reported in Supplemental figure 6A. (TIF) [file ppat.1002408.s007.tif]

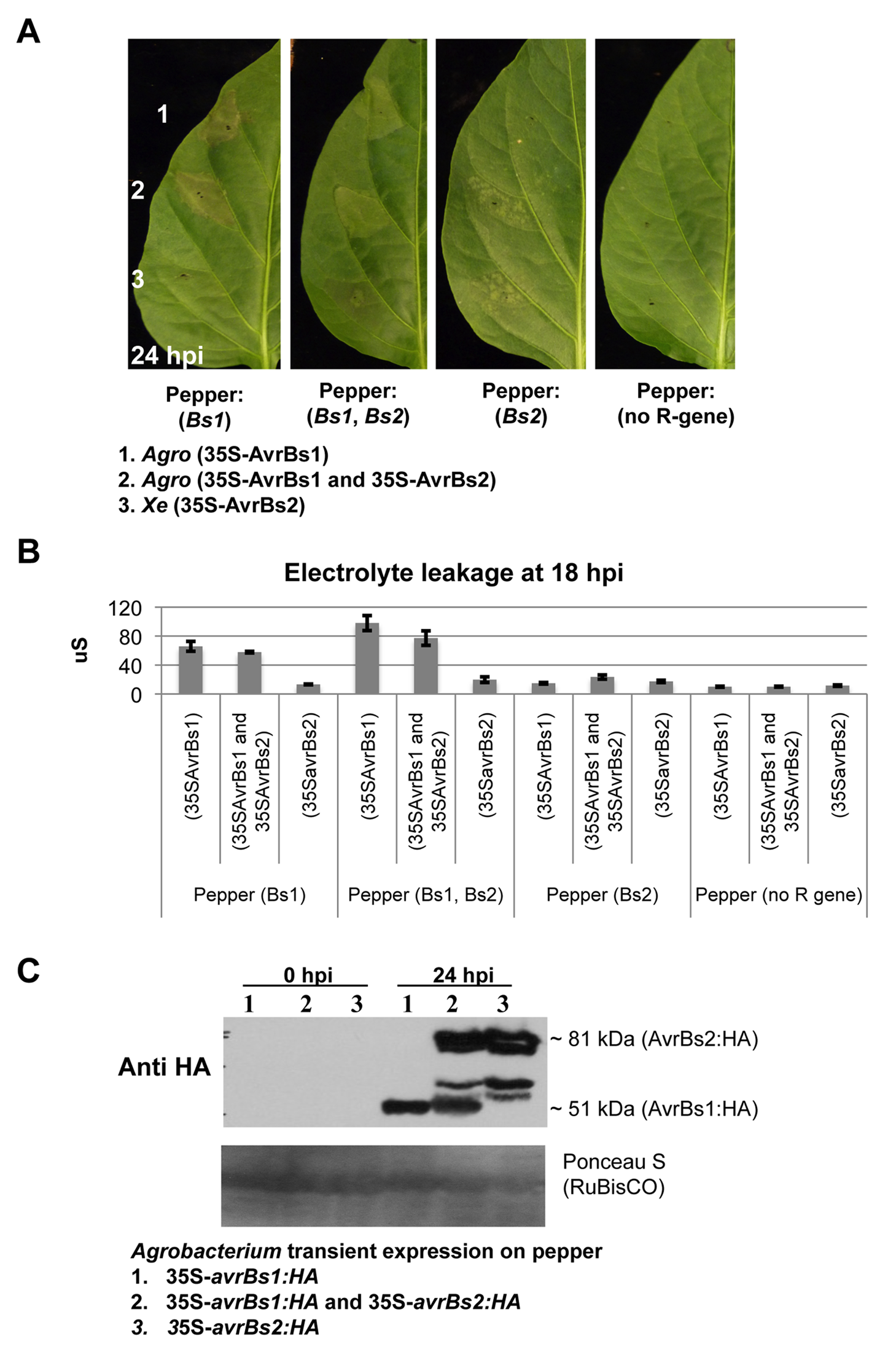

Supplement: Supplemental Figure S8 — Bs2 activation dependent suppression of the AvrBs1/Bs1 fast HR phenotype not observed when expressed inside plant cells via Agrobacterium transient expression. A. Agrobacterium transient expression strains containing 1. Agro 35S-avrBs1, 2. Agro 35S-avrBs1 + 35S-avrBs2 and 3. Agro 35S-AvrBs2 were inoculated on pepper plants containing the Bs1, (Bs1 and Bs2) and no resistance genes. During the co-expression of AvrBs2 and AvrBs1 no epistasis was observed as the phenotype of the Bs1 HR was not altered by the co-expression of AvrBs2. B. Electrolyte leakage was observed for the same combinations as shown in panel A. The electrolyte leakage phenotype of the Bs1 HR was observed when both AvrBs2 and AvrBs1 were co-expressed in Agro confirming that there was no epistasis when the genes are co-expressed in planta. C. Immunoblot detection of AvrBs2-HA and AvrBs1-HA expressed in pepper plants via Agrobacterium transient expression at 0 and 24 hpi. This result showed that the activation of Bs2-specified resistance did not interfere with the detection of the Bs1 protein. (TIF) [file ppat.1002408.s008.tif]

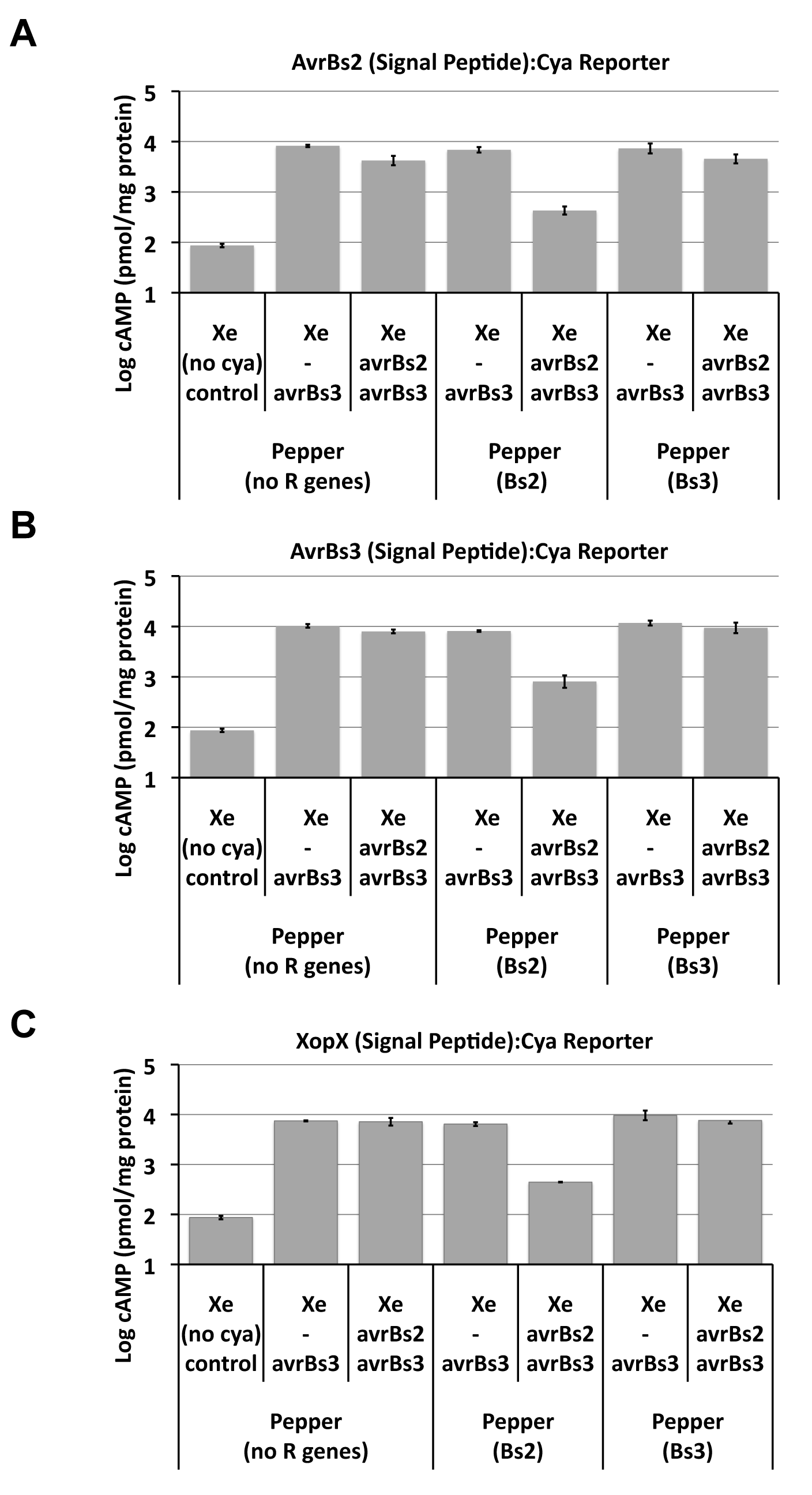

Supplement: Supplemental Figure S9 — Bs2 activation by AvrBs2 blocked subsequent TTSS delivery of multiple effectors to host cells. TTSS effector delivery reporter constructs consisted of the effector promoter and the secretion and translocation signal peptides translationally fused to adenylate cyclase (Cya). Pepper plants (no R genes, Bs2 or Bs3) were sampled 8 hours post-inoculation to avoid in planta multiplication of the reporter strain pairs (with and without avrBs2) and assayed for cyclic AMP (cAMP). Effector-Cya translational fusion and calmodulin from the plant cell led to elevated levels of cAMP. A. AvrBs21-212-Cya reporter in Xanthomonas strains GM98-38-1 Xe (avrBs2 and avrBs3) and Xe (avrBs3) inoculated onto pepper plants (no R genes, Bs3 or Bs2). In planta cAMP levels were assayed. Student t-test was used to compare TTSS delivery of effector reporter in an Xe strain with and without avrBs2 on Bs2 pepper plants; p-values were <0.01. B. AvrBs31-212-Cya reporter in Xanthomonas strains GM98-38-1 Xe (avrBs2 and avrBs3) and Xe (avrBs3) inoculated onto pepper plants (no R genes, Bs3 or Bs2). In planta cAMP levels were assayed. Student t-test was used to compare TTSS delivery of effector reporter in an Xe strain with and without avrBs2 on Bs2 pepper plants; p-values were <0.01. C. XopX1-183-Cya reporter in Xanthomonas strains GM98-38-1 Xe (avrBs2 and avrBs3) and Xe (avrBs3) inoculated onto pepper plants (no R genes, Bs3 or Bs2). In planta cAMP levels were assayed. Student t-test was used to compare TTSS delivery of effector reporter in an Xe strain with and without avrBs2 on Bs2 pepper plants; p-values were <0.01. (TIF) [file ppat.1002408.s009.tif]

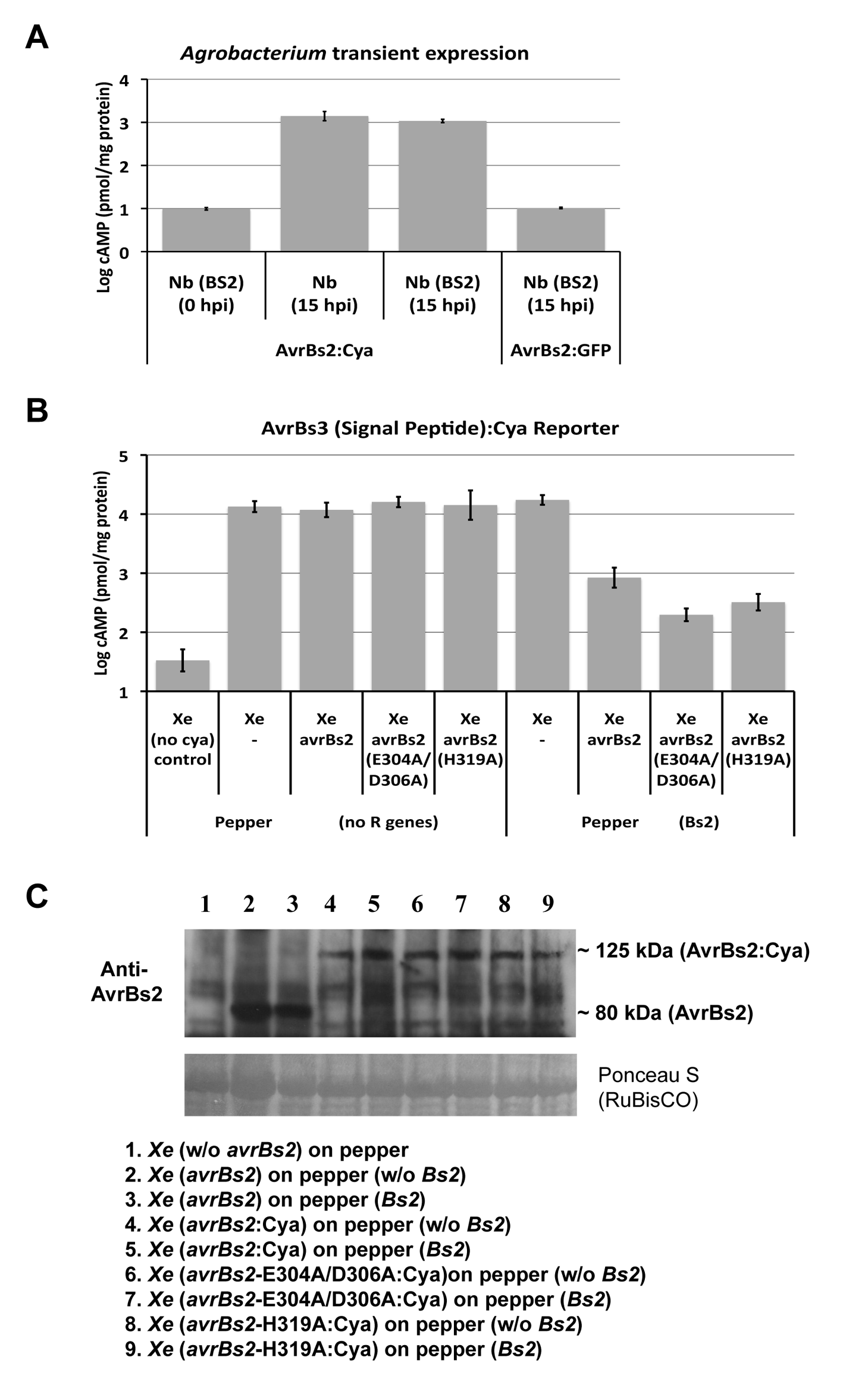

Supplement: Supplemental Figure S10 — Elevated levels of in-planta cyclic AMP resulting from Agrobacterium transient expression of 35S- avrBs2 :Cya was not blocked by Bs2 activation. Also avrBs2 GDE catalytic mutations were still able to block subsequent TTSS effector delivery to pepper host. Also Bs2 activation does not change effector protein levels in Xe. A. Agrobacterium transient expression of 35S-avrBs2:Cya in Nicotiana benthamiana with and without Bs2 sampled at 15 hpi were similar for elevated levels of cyclic AMP. B. AvrBs3 (signal peptide):Cya TTSS effector reporter recombined into Xanthomonas strains GM98-38-1 Xe (avrBs2, avrBs2-E304A/D306A or avrBs2-H319A) had similar reduced levels of cyclic AMP in the presence of Bs2 compared to pepper host without Bs2. C. Immunoblot assays of high titer inoculation (5×10^8^ CFU/ml) of pepper (w/o Bs2) and pepper (Bs2) at 8 hpi with Xe (avrBs2), Xe (avrBs2-Cya), Xe (avrBs2-E304A/D306A:Cya) and Xe (avrBs2-H319A:Cya) detected no reductions of protein levels associated with Bs2 activation. (TIF) [file ppat.1002408.s010.tif]
